# Supplementary material for: Intergenic risk variant rs56258221 skews the fate of naive CD4+ T cells via miR4464-BACH2 interplay in primary sclerosing cholangitis
Source: Cell Rep Med. 2024 Jun 19;5(7):101620. doi: 10.1016/j.xcrm.2024.101620 (PMC11293351; doi:10.1016/j.xcrm.2024.101620)
Supplement: Document S1. Figures S1–S3 and Tables S1–S7 [file mmc1.pdf]

Cell Reports Medicine, Volume *5*

Supplemental information

Intergenic risk variant *rs56258221* skews the fate of naive CD4^{+} T cells via miR4464-BACH2 interplay in primary sclerosing cholangitis

Tobias Poch, Jonas Bahn, Christian Casar, Jenny Krause, Ioannis Evangelakos, Hilla Gilladi, Lilly K. Kunzmann, Alena Laschtowitz, Nicola Iuso, Anne-Marie Schäfer, Laura A. Liebig, Silja Steinmann, Marcial Sebode, Trine Folseraas, Lise K. Engesæter, Tom H. Karlsen, Andre Franke, Norbert Hubner, Christian Schlein, Eithan Galun, Samuel Huber, Ansgar W. Lohse, Nicola Gagliani, Dorothee Schwinge, and Christoph Schramm

SUPPLEMENTARY FIGURES AND FIGURE LEGENDS

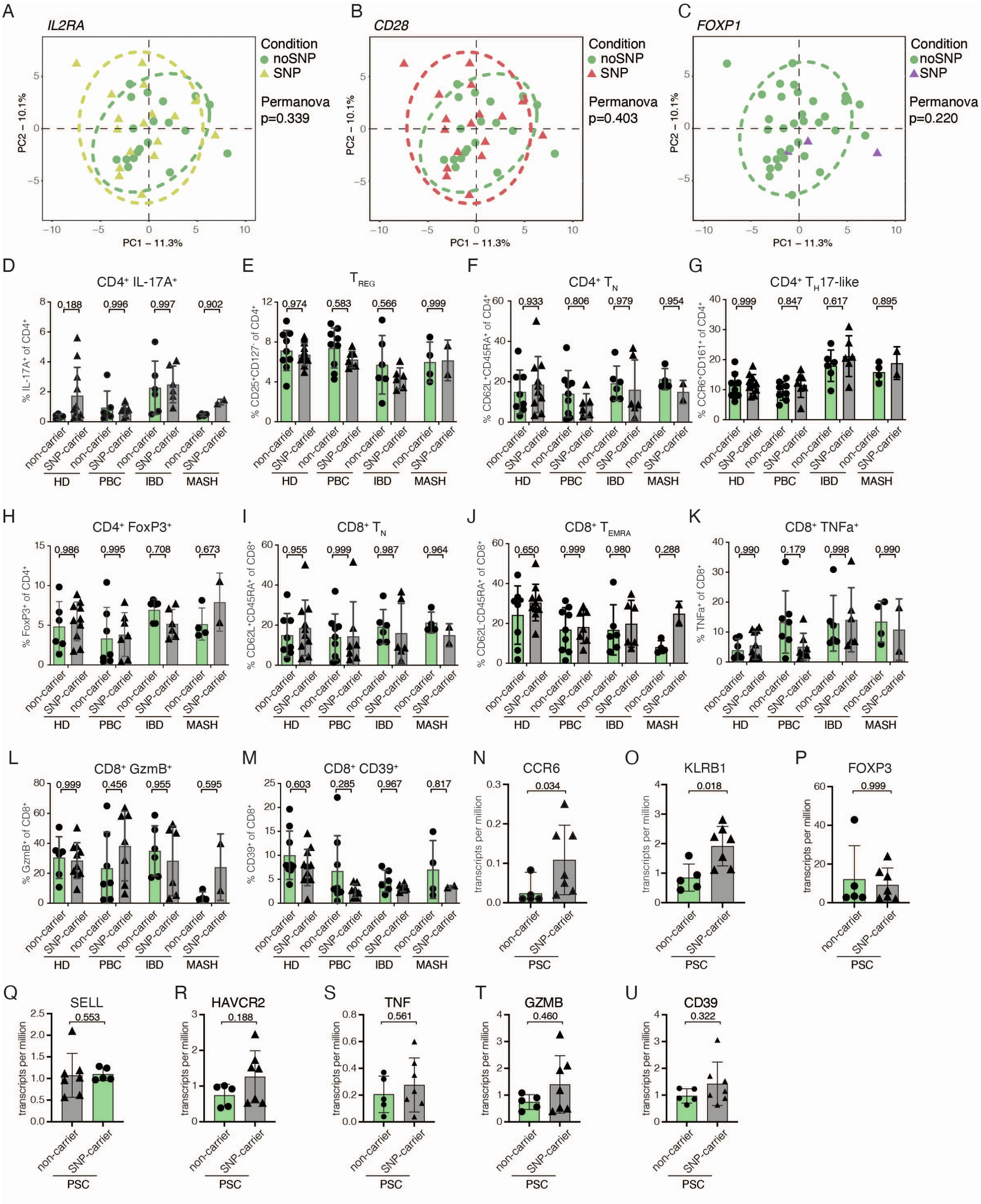

Fig. S1: People with PSC carrying *rs56258221* show distinct immunophenotypes. Related to Figure 1.

(**A-C**) Principal component analyses of the analyzed immunophenotyping data from peripheral blood of people with PSC, separated by the genotype for polymorphism *rs4147359* (*IL2RA*) (A), *rs7426056* (*CD28*) (B) and *rs80060485* (*FOXP1*) (C). (**D-H**) Frequencies of different $CD4^{+}$ T cell subsets. (**D**) T_N, identified by CD62L/CD45RA. (**E**) T_H17 cells identified by IL-17A expression upon stimulation with PMA/Ionomycin. (**F**)

T_H17 cells identified by CCR6/CD161. (**G**) T_{REG} identified by CD127/CD25. (**H**) T_{REG} identified by FoxP3 expression upon stimulation with PMA/Ionomycin. (**I**-**M**) Frequencies of different $CD8^+$ T cell subsets. (**I**) T_N, identified by CD62L/CD45RA. (**J**) T_{EMRA}, identified by CD62L/CD45RA. (**K**) CD39 expression on $CD8^+$ T cells. (**L**) TNFa expression upon stimulation with PMA/Ionomycin. (**M**) GzmB expression upon stimulation with PMA/Ionomycin. Statistics: Normality distribution was tested by Kolmogorov-Smirnov test; normal distribution: Welch's t-Test or 2-way ANOVA; no normal distribution: Mann-Whitney U Test. $p<0.05$ was considered statistically significant. Data is presented as mean ± SD and deriving from $n \geq 2$ repeats per experiment.

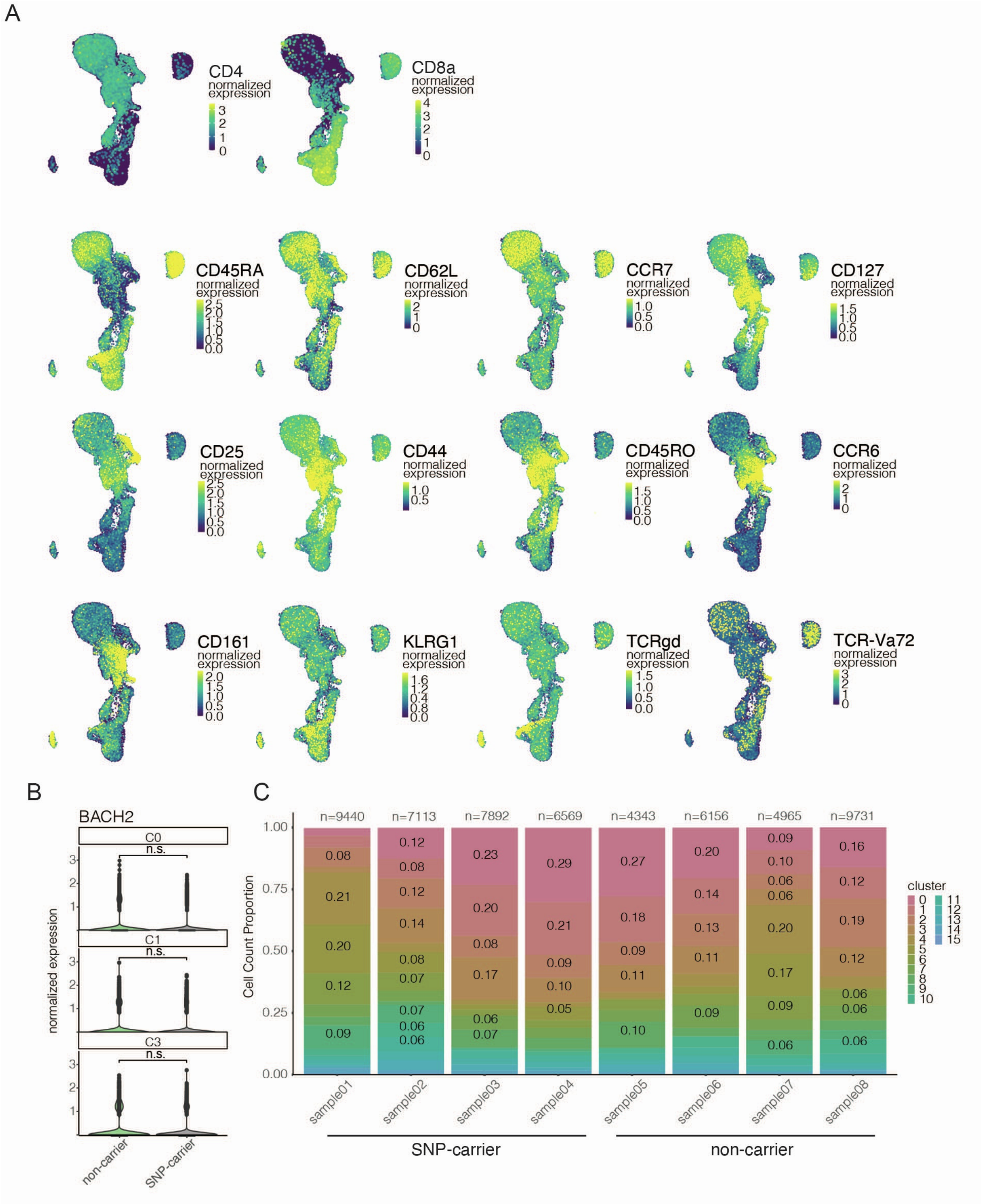

Fig. S2: Characterization of peripheral blood T cells from homozygous carriers of *rs56258221*. Related to Figure 2.

(**A**) Data of antibody-derived tags, resembling protein expression, mapped onto the UMAP from Fig. 2B. Differentiation markers were used to assist cluster assignment. (**B**) BACH2 mRNA expression levels in $CD4^+$ (C0, C1) and $CD8^+$ T_N (C3) between SNP-carriers and non-carriers. (**C**) Distribution of cells per sample across the different clusters of T cells between SNP-carriers and non-carriers.

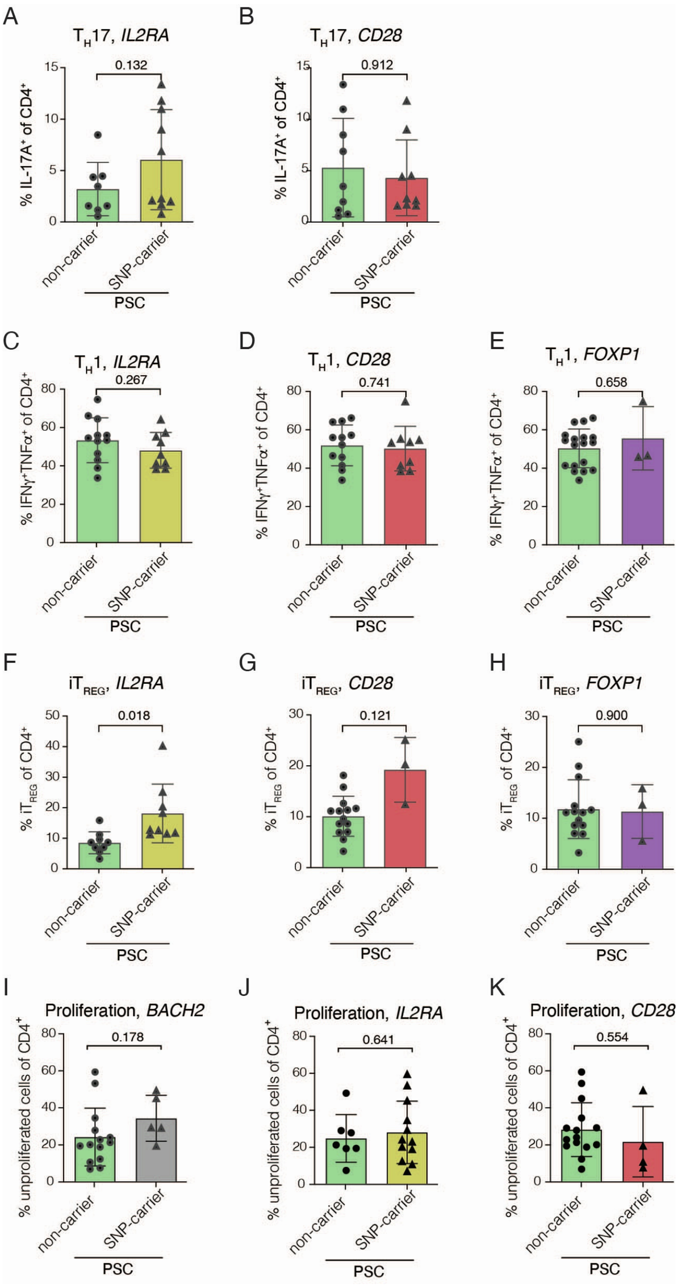

Fig. S3: Skewed differentiation of naive CD4$^+$ T cells from people with PSC is associated with *rs56258221*. Related to Figure 3.

(**A-B**) After 12 d of polarizing culture of CD4$^+$ T$_N$ from patients with PSC (n=18) the frequency of CD4$^+$IL-17A$^+$ cells was determined and separated by *rs4147359 (IL2RA)* (A), *rs7426056 (CD28)* (B). *rs80060485 (FOXP1)* is not shown as no datapoints for SNP-carriers were recordfed. (**C-E**) After 7 d of polarizing culture of CD4$^+$ T$_N$ from patients with PSC (n=21), the frequency of T CD4$^+$CXCR3$^+$TBET$^+$IFNg$^+$TNFa$^+$ cells was determined and data was separated in to carriers and non-carriers of *rs4147359 (IL2RA)* (C), *rs7426056 (CD28)* (D) and *rs80060485 (FOXP1)* (E). (**F-H**) After 7 days of polarizing culture of CD4$^+$ T$_N$ from patients with PSC (n=18), the frequencies of CD4$^+$CD25$^+$CD152$^+$FOXP3$^+$ cells were determined and data was separated into

carriers and non-carriers of *rs4147359 (IL2RA)* (F), *rs7426056 (CD28)* (G) and *rs80060485 (FOXP1)* (H). (**I-K**) After 6 d of non-polarizing culture of *CellTrace Violet*-marked $CD4^+$ T_N from patients with PSC, the frequency of divided and undivided cells was determined by flow cytometry. Data was separated into carriers and non-carriers of *rs56258221* (*BACH2*) *rs4147359* (*IL2RA*) (J), *rs7426056* (*CD28*) (K). Data is presented as mean ± SD and deriving from n ≥ 2 repeats per experiment.

SUPPLEMENTARY TABLES

Table S1: Clinical parameters of people with PSC, related to Figure 1A-M.

patient #	SNP-status	gender	year of birth	IBD status	other autoimmune disease	immunosuppr. therapy	transient elastography [kPa]	MELD-score	ANAs (Ref.: <1:80)	SMA (Ref.: <1:80)	SLA/LP (Ref.: <20relU/ml)
**patient #1**	non-carrier	cis	1967	PSC-assoc. colitis	/	/	10	6,0	negative	negative	negative
**patient #2**	non-carrier	cis	1968	PSC-assoc. colitis	Hashimoto's disease	/	8,8	7,1	1:80	negative	negative
**patient #3**	non-carrier	cis	1983	/	/	/	4,5	6,8	negative	negative	negative
**patient #4**	non-carrier	cis	1957	PSC-assoc. colitis	/	/	3,9	6,0	1:80	negative	negative
**patient #5**	non-carrier	cis	1968	/	/	/	5,3	6,0	negative	n.a.	n.a.
**patient #6**	non-carrier	cis	1982	PSC-assoc. colitis	/	/	60,4	33,4	1:640	negative	negative
**patient #7**	non-carrier	cis	1968	PSC-assoc. colitis	/	/	4,7	6,0	1:80	negative	negative
**patient #8**	non-carrier	cis	1965	/	/	/	16,5	15,8	1:160	negative	negative
**patient #9**	non-carrier	cis	1955	/	/	/	6,3	6,0	negative	negative	negative
**patient #10**	non-carrier	cis	1959	/	/	/	5,7	6,0	1:80	negative	negative
**patient #11**	non-carrier	cis	1965	PSC-assoc. colitis	/	Azathioprine	32	6,9	1:640	1:160	negative
**patient #12**	non-carrier	cis	1967	PSC-assoc. colitis	/	/	4,9	6,7	1:320	negative	negative
**patient #13**	non-carrier	cis	1978	PSC-assoc. colitis	Graves' disease	/	4,7	6,0	1:160	1:80	negative
**patient #14**	non-carrier	cis	1977	PSC-assoc. colitis	/	Azathioprine	4	6,2	negative	negative	negative
**patient #15**	non-carrier	cis	1974	PSC-assoc. colitis	/	/	3,7	6,0	1:320	negative	negative
**patient #16**	non-carrier	cis	1982	PSC-assoc. colitis	/	/	4,3	6,0	1:320	negative	negative
**patient #17**	non-carrier	cis	1941	/	/	/	4,1	6,0	negative	negative	negative
**patient #18**	non-carrier	cis	1966	/	/	/	7,8	6,0	1:160	negative	negative
**patient #19**	heterozygous	cis	1950	PSC-assoc. colitis	/	/	7,6	6,0	1:1280	negative	negative
**patient #20**	heterozygous	cis	1979	PSC-assoc. colitis	/	/	34,4	13,0	1:5120	1:180	negative
**patient #21**	heterozygous	cis	1979	PSC-assoc. colitis	/	/	11,7	6,0	1:320	negative	negative
**patient #22**	heterozygous	cis	1953	PSC-assoc. colitis	/	/	3,5	6,0	1:320	negative	negative
**patient #23**	heterozygous	cis	1986	PSC-assoc. colitis	/	/	75	7,2	1:80	negative	negative
**patient #24**	heterozygous	cis	1978	PSC-assoc. colitis	/	/	4,4	6,0	1:320	1:320	negative
**patient #25**	heterozygous	cis	1958	/	/	/	n.a.	18,9	1:160	1:80	negative
**patient #26**	heterozygous	cis	1963	/	/	/	70	14,5	1:320	1:80	negative
**patient #27**	heterozygous	cis	1957	PSC-assoc. colitis	Hashimoto's disease	/	7,2	6,0	1:80	1:80	negative
**patient #28**	heterozygous	cis	1976	/	/	/	11,3	7,8	1:160	negative	negative
**patient #29**	heterozygous	cis	1969	PSC-assoc. colitis	DM type 1	/	6,6	7,7	1:320	1:160	negative
**patient #30**	heterozygous	cis	1952	PSC-assoc. colitis	/	/	n.a.	9,6	1:160	negative	negative
**patient #31**	heterozygous	cis	1977	PSC-assoc. colitis	/	/	4,8	6,0	1:180	negative	negative
**patient #32**	heterozygous	cis	1969	/	/	/	3,6	6,0	1:160	negative	negative
**patient #33**	heterozygous	cis	1956	Crohn's disease	Graves' disease	/	n.a.	21,1	1:640	negative	n.a.
**patient #34**	homozygous	cis	1957	PSC-assoc. colitis	/	/	7,1	7,5	1:640	negative	negative
**patient #35**	homozygous	cis	1969	PSC-assoc. colitis	/	/	6	6,8	1:1280	negative	negative
**patient #36**	homozygous	cis	1963	PSC-assoc. colitis	/	/	37,4	6,0	1:640	negative	negative

Table S2: Clinical parameters of people with PSC, related to Figure 2A-J.

patient #	SNP-status	gender	year of birth	IBD status	other autoimmune disease	immunosuppr. therapy	transient elastography [kPa]	MELD-Score	ANAs (Ref.: <1:80)	SMA (Ref.: <1:80)	SLA/LP (Ref.: <20relU/ml)
**patient #1**	homozygous	cis	1969	PSC-assoc. colitis	/	/	6	6,8	1:1280	negative	negative
**patient #2**	homozygous	cis	1987	/	/	/	20	11,8	1:320	negative	negative
**patient #3**	homozygous	cis	1980	PSC-assoc. colitis	/	Vedolizumab	5,6	6,0	negative	negative	negative
**patient #4**	homozygous	cis	1975	PSC-assoc. colitis	/	/	5,3	6,0	negative	1:80	negative
**patient #5**	non-carrier	cis	1966	/	/	/	7,8	6,0	1:160	negative	negative
**patient #6**	non-carrier	cis	1979	/	/	/	4,7	6,0	1:160	negative	negative
**patient #7**	non-carrier	cis	1976	/	/	/	6,3	6,0	1:160	negative	negative
**patient #8**	non-carrier	cis	1972	Crohn's disease	/	/	6,7	6,0	1:160	negative	negative

Table S3: Clinical parameters of people with PSC, related to Figures 2K-L and 4D.

patient #	SNP-status	gender	year of birth	IBD status	other autoimmune disease	immunosuppr. therapy	transient elastography [kPa]	MELD-Score	ANAs (Ref.: <1:80)	SMA (Ref.: <1:80)	SLA/LP (Ref.: <20relU/ml)
**patient #1**	non-carrier	cis	1966	/	/	/	7,8	6,0	1:160	negative	negative
**patient #2**	non-carrier	cis	1979	/	/	/	4,7	6,0	1:160	negative	negative
**patient #3**	non-carrier	cis	1976	/	/	/	6,3	6,0	1:160	negative	negative
**patient #4**	non-carrier	cis	1972	Crohn's disease	/	/	6,7	6,0	1:160	negative	negative
**patient #5**	non-carrier	cis	1980	PSC-assoc. colitis	/	/	7,8	6,0	1:160	negative	negative
**patient #6**	non-carrier	cis	1994	PSC-assoc. colitis	/	Azathioprin	4,9	6,0	1:320	1:80	negative
**patient #7**	non-carrier	cis	1975	PSC-assoc. colitis	Psoriasis vulgaris	Ustekinumab	6,3	6,0	1:160	negative	negative
**patient #8**	homozygous	cis	1944	PSC-assoc. colitis	/	/	14,3	14,1	1:160	1:80	negative
**patient #9**	homozygous	cis	1957	PSC-assoc. colitis	/	/	7,1	7,5	1:640	negative	negative
**patient #10**	homozygous	cis	1986	PSC-assoc. colitis	/	/	5,2	6,0	1:640	1:80	negative
**patient #11**	homozygous	cis	1972	PSC-assoc. colitis	/	/	43,8	14,5	1:320	negative	negative
**patient #12**	homozygous	cis	1963	PSC-assoc. colitis	/	/	37,4	6,0	1:640	negative	negative
**patient #13**	homozygous	cis	1969	PSC-assoc. colitis	/	/	6	6,8	1:1280	negative	negative
**patient #14**	homozygous	cis	1987	/	/	/	20	11,8	1:320	negative	negative
**patient #15**	homozygous	cis	1980	PSC-assoc. colitis	/	Vedolizumab	5,6	6,0	negative	negative	negative
**patient #16**	homozygous	cis	1975	PSC-assoc. colitis	/	/	5,3	6,0	negative	1:80	negative

Table S4: Clinical parameters of people with PSC, related to Figure 3H+K.

patient #	SNP-Status	gender	year of birth	IBD status	other autoimmune disease	immunosuppr. therapy	transient elastography [kPa]	MELD-score	ANAs (Ref.: <1:80)	SMA (Ref.: <1:80)	SLA/LP (Ref.: <20relU/ml)
**patient #1**	non-carrier	cis	1982	PSC-assoc. colitis	/	/	4,3	6,0	1:320	negative	negative
**patient #2**	non-carrier	cis	1977	PSC-assoc. colitis	/	/	6	6,0	1:80	1:80	negative
**patient #3**	non-carrier	cis	1967	PSC-assoc. colitis	/	/	4,9	6,7	1:320	negative	negative
**patient #4**	non-carrier	cis	1967	PSC-assoc. colitis	/	/	6,1	6,0	1:160	negative	negative
**patient #5**	non-carrier	cis	1980	PSC-assoc. colitis	/	/	2,1	9,9	1:640	negative	negative
**patient #6**	non-carrier	cis	1977	PSC-assoc. colitis	/	/	7,6	6,0	1:160	negative	negative
**patient #7**	non-carrier	cis	1972	PSC-assoc. colitis	/	/	23	6,0	negative	negative	negative
**patient #8**	non-carrier	cis	1987	/	/	/	75	6,0	negative	negative	negative
**patient #9**	non-carrier	cis	1964	/	/	/	4,7	6,0	1:160	negative	negative
**patient #10**	non-carrier	cis	1975	PSC-assoc. colitis	/	Azathioprin	14	7,1	1:160	negative	23.0
**patient #11**	non-carrier	cis	1976	PSC-assoc. colitis	Hashimoto's disease	/	7,4	6,0	1:80	negative	negative
**patient #12**	heterozygous	cis	1964	PSC-assoc. colitis	/	/	4,8	6,9	1:320	negative	negative
**patient #13**	heterozygous	cis	1950	PSC-assoc. colitis	/	/	7,6	6,0	1:1280	negative	negative
**patient #14**	heterozygous	cis	1961	PSC-assoc. colitis	Rheumatoid arthritis	Baricitinib	12,9	6,0	1:160	negative	negative
**patient #15**	heterozygous	cis	1980	PSC-assoc. colitis	/	/	6,1	6,0	1:80	negative	negative
**patient #16**	heterozygous	cis	1953	PSC-assoc. colitis	/	/	3,5	6,0	1:320	negative	negative
**patient #17**	homozygous	cis	1963	PSC-assoc. colitis	/	/	37,4	6,0	1:640	negative	negative
**patient #18**	homozygous	cis	1994	PSC-assoc. colitis	/	/	71	6,6	1:640	1:160	negative
**patient #19**	homozygous	cis	1980	PSC-assoc. colitis	/	/	5,6	6,0	negative	negative	negative

Table S5: Clinical parameters of people with PSC, related to Figure 3G+J.

patient #	SNP-status	gender	year of birth	IBD status	other autoimmune disease	immunosuppr. therapy	transient elastography [kPa]	MELD-score	ANAs (Ref.: <1:80)	SMA (Ref.: <1:80)	SLA/LP (Ref.: <20relU/ml)
**patient #1**	non-carrier	cis	1997	Crohn's disease	/	/	7	8,3	1:160	negative	negative
**patient #2**	non-carrier	cis	1956	/	/	/	4,5	6,0	1:160	negative	negative
**patient #3**	non-carrier	cis	1962	PSC-assoc. colitis	/	/	5,6	6,0	1:80	1:160	negative
**patient #4**	non-carrier	cis	1980	PSC-assoc. colitis	/	/	2,1	9,9	1:640	negative	negative
**patient #5**	non-carrier	cis	1951	PSC-assoc. colitis	/	Vedolizumab	4,3	6,0	1:160	negative	negative
**patient #6**	non-carrier	cis	1974	PSC-assoc. colitis	/	/	3,7	6,0	1:320	negative	negative
**patient #7**	non-carrier	cis	1950	/	/	/	4,6	6,0	1:5120	negative	negative
**patient #8**	non-carrier	cis	1980	PSC-assoc. colitis	/	/	7,8	6,0	1:160	negative	negative
**patient #9**	non-carrier	cis	1987	PSC-assoc. colitis	/	/	5,8	6,0	1:640	1:160	negative
**patient #10**	non-carrier	cis	1987	PSC-assoc. colitis	/	/	6,1	6,0	1:160	negative	negative
**patient #11**	non-carrier	cis	1962	PSC-assoc. colitis	/	/	3,3	6,0	1:80	negative	negative
**patient #12**	heterozygous	cis	1999	PSC-assoc. colitis	/	Fligotinib	4,7	6,0	1:80	negative	negative
**patient #13**	heterozygous	cis	1977	PSC-assoc. colitis	/	/	4,8	6,0	1:180	negative	negative
**patient #14**	heterozygous	cis	1966	/	Hashimoto's disease	/	4,2	6,0	1:160	negative	negative
**patient #15**	heterozygous	cis	1988	PSC-assoc. colitis	/	/	7,8	6,0	1:160	negative	negative
**patient #16**	heterozygous	cis	1968	PSC-assoc. colitis	/	/	42	16,0	1:5120	negative	n.a.
**patient #17**	heterozygous	cis	1957	/	/	/	6,6	6,0	1:160	negative	negative
**patient #18**	heterozygous	cis	1976	PSC-assoc. colitis	/	/	4,9	6,0	1:80	negative	negative
**patient #19**	heterozygous	cis	2000	Crohn's disease	/	Ustekinumab	3,7	6,0	1:1280	negative	negative
**patient #20**	heterozygous	cis	1969	/	/	/	3,6	6,0	1:160	negative	negative
**patient #21**	heterozygous	cis	1977	/	/	/	4,9	7,5	1:160	negative	negative

Table S6: Clinical parameters of people with PSC, related to Figure 3F+I.

patient #	SNP-status	gender	year of birth	IBD status	other autoimmune disease	immunosuppr. therapy	transient elastography [kPa]	MELD-score	ANAs (Ref.: <1:80)	SMA (Ref.: <1:80)	SLA/LP (Ref.: <20relU/ml)
**patient #1**	non-carrier	cis	1988	PSC-assoc. colitis	/	/	28	6,0	1:80	1:160	negative
**patient #2**	non-carrier	cis	1961	Crohn's disease	Hashimoto's disease	/	4,7	6,0	1:320	negative	negative
**patient #3**	non-carrier	cis	1977	PSC-assoc. colitis	Hashimoto's disease	/	10,4	7,4	1:640	negative	negative
**patient #4**	non-carrier	cis	1963	/	/	/	6,2	6,0	1:80	1:80	negative
**patient #5**	non-carrier	cis	1979	/	/	/	4,7	6,0	1:160	negative	negative
**patient #6**	non-carrier	cis	1988	Crohn's disease	/	Vedolizumab	8,8	12,8	1:640	negative	negative
**patient #7**	heterozygous	cis	1967	/	/	/	9,3	6,0	1:160	negative	negative
**patient #8**	heterozygous	cis	1975	PSC-assoc. colitis	/	/	7,1	6,0	1:160	negative	negative
**patient #9**	heterozygous	cis	1976	/	/	/	7,4	6,0	1:320	negative	negative
**patient #10**	heterozygous	cis	1976	PSC-assoc. colitis	/	/	4,9	6,0	1:80	negative	negative
**patient #11**	heterozygous	cis	1950	/	/	/	9,3	6,0	1:160	negative	negative
**patient #12**	heterozygous	cis	1950	PSC-assoc. colitis	/	/	12,7	7,2	1:320	negative	negative
**patient #13**	heterozygous	cis	1965	/	/	/	6,7	6,0	1:160	negative	negative
**patient #14**	heterozygous	cis	1965	/	/	/	5	6,0	1:320	negative	negative
**patient #15**	heterozygous	cis	1959	PSC-assoc. colitis	/	/	12,3	6,0	1:5120	negative	negative
**patient #16**	homozygous	cis	1963	PSC-assoc. colitis	/	/	37,4	6,0	1:640	negative	negative
**patient #17**	homozygous	cis	1944	PSC-assoc. colitis	/	/	14,3	14,1	1:160	1:80	negative

*Abbreviations:

n.a.: information not available

/ : patient without IBD; no immunosuppressive therapy; absence of tested autoantibodies

Table S7: Primers for generation of BACH2 plasmid, related to Figure 5F-H.

Primer	Sequence	Gene
Pr1	CACACGAGCTCGATACTGTACAGTAATGG	Forward primer (3'UTR BACH2-mRNA)
Pr2	CACACGTCGACAAAGCAAAGAATATACAG	Reverse primer (3'UTR BACH2-mRNA)
Fr1	(SacI)GAGCTCGATACTGTACANCCACTGGTCAACTTTGCCACTTGCA CTGAGTTTTGGGT**CAAACCTA**TTTTCTTAAATGAAGTTGTAACTTCG GTATAACTCAAGTATACTGTATATTCTTTGCTTTGTCGAC(SalI)	BACH2 3'UTR fragment (miR4464 site in bold letters)
Pr4	GAAAATACTCGAGACCCAAAACTCAGTGC	Reverse primer (to mutate the miR4464 site)
Fr2	(SacI)GAGCTCGATACTGTACANCCACTGGTCAACTTTGCCACTTGCA CTGAGTTTTGGGT**CTCGAGTA**TTTTCTTAAATGAAGTTGTAACTTCG GTATAACTCAAGTATACTGTATATTCTTTGCTTTGTCGAC(SalI)	Mutated miR-4464 site in BACH2 3'-UTR (mutated part in bold letters)
