# Supplementary material for: Intergenic risk variant rs56258221 skews the fate of naive CD4+ T cells via miR4464-BACH2 interplay in primary sclerosing cholangitis
Source: Cell Rep Med. 2024 Jun 19;5(7):101620. doi: 10.1016/j.xcrm.2024.101620 (PMC11293351; doi:10.1016/j.xcrm.2024.101620)
Supplement: Document S2. Article plus supplemental information [file mmc2.pdf]

Cell Reports Medicine

Article

Intergenic risk variant *rs56258221* skews the fate of naive $CD4^+$ T cells via miR4464-BACH2 interplay in primary sclerosing cholangitis

Graphical abstract

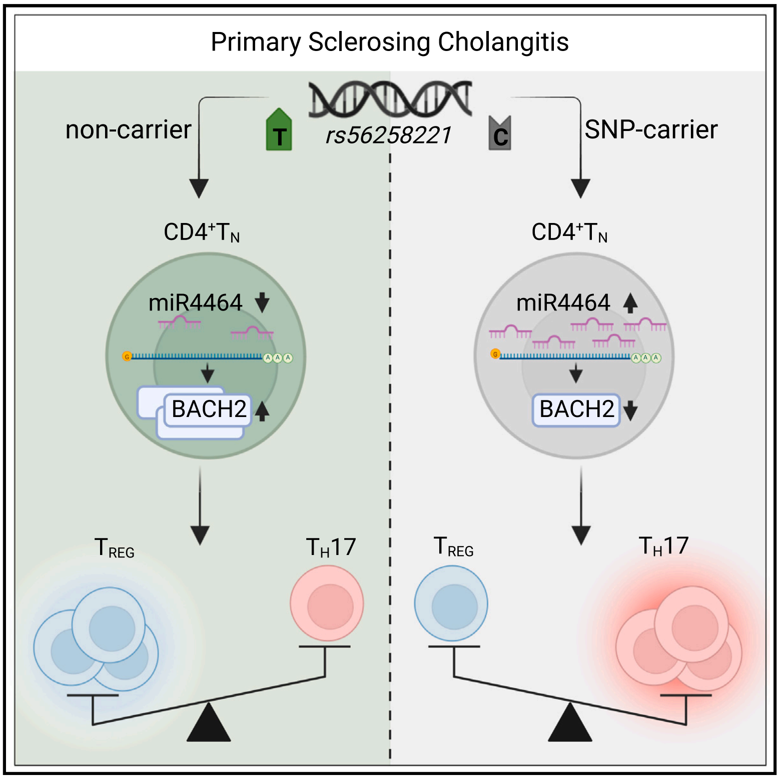

Highlights

- People with PSC carrying *rs56258221* bear a pro-inflammatory immunophenotype
- *rs56258221* impairs BACH2 expression and skews differentiation of naive $CD4^+$ T cells
- Aberrant $CD4^+$ T_N differentiation is linked to elevated BACH2-targeting miR4464 levels
- Carriers of *rs56258221* show clinical signs of accelerated disease progression

Authors

Tobias Poch, Jonas Bahn, Christian Casar, ..., Nicola Gagliani, Dorothee Schwinge, Christoph Schramm

Correspondence

c.schramm@uke.de

In brief

Poch, Bahn et al. identify the primary sclerosing cholangitis-associated genetic polymorphism *rs56258221* to enhance miR4464 expression in naive $CD4^+$ T cells. By targeting BACH2, a gatekeeper of T cell quiescence, elevated miR4464-levels change the fate of naive $CD4^+$ T cells towards pro-inflammatory subsets.

Poch et al., 2024, Cell Reports Medicine 5, 101620
July 16, 2024 © 2024 The Authors. Published by Elsevier Inc.
https://doi.org/10.1016/j.xcrm.2024.101620

CellPress

Cell Reports Medicine

CellPress
OPEN ACCESS

Article

Intergenic risk variant *rs56258221* skews the fate of naive CD4+ T cells via miR4464-BACH2 interplay in primary sclerosing cholangitis

Tobias Poch,[1,17,18] Jonas Bahn,[1,18] Christian Casar,[1,2] Jenny Krause,[1,3] Ioannis Evangelakos,[4] Hilla Gilladi,[5] Lilly K. Kunzmann,[1] Alena Laschtowitz,[1,6] Nicola Iuso,[1] Anne-Marie Schäfer,[1] Laura A. Liebig,[1,7] Silja Steinmann,[1,3] Marcial Sebode,[1,3] Trine Folseraas,[3,8,9] Lise K. Engesæter,[3,8,9] Tom H. Karlsen,[3,8,9] Andre Franke,[10] Norbert Hubner,[7,11,12] Christian Schlein,[4] Eithan Galun,[5] Samuel Huber,[1,3,13] Ansgar W. Lohse,[1,3,13] Nicola Gagliani,[1,13,14,15] Dorothee Schwinge,[1,19] and Christoph Schramm[1,3,13,16,19,20,*]

[1]I. Department of Medicine, University Medical Centre Hamburg-Eppendorf, 20246 Hamburg, Germany
[2]Bioinformatics Core, University Medical Centre Hamburg-Eppendorf, 20246 Hamburg, Germany
[3]European Reference Network for Hepatological Diseases (ERN RARE-LIVER), 20246 Hamburg, Germany
[4]Institute of Human Genetics, University Medical Centre Hamburg-Eppendorf, 20246 Hamburg, Germany
[5]The Goldyne-Savad Institute of Gene Therapy, Hadassah Hebrew University Hospital, Jerusalem 91120, Israel
[6]Department of Hepatology and Gastroenterology, Charité Universitätsmedizin Berlin, 13353 Berlin, Germany
[7]Cardiovascular and Metabolic Sciences, Max Delbrück Centre for Molecular Medicine in the Helmholtz Association (MDC), 13125 Berlin, Germany
[8]Norwegian PSC Research Centre, Department of Transplantation Medicine, Oslo University Hospital Rikshospitalet, 0372 Oslo, Norway
[9]Research Institute of Internal Medicine, Oslo University Hospital Rikshospitalet, 0372 Oslo, Norway
[10]Institute of Clinical Molecular Biology, Christian-Albrechts-University of Kiel, 24105 Kiel, Germany
[11]DZHK (German Centre for Cardiovascular Research), Partner Site Berlin, 10785 Berlin, Germany
[12]Charité-Universitätsmedizin Berlin, 10117 Berlin, Germany
[13]Hamburg Centre for Translational Immunology, University Medical Centre Hamburg-Eppendorf, 20246 Hamburg, Germany
[14]Department for General, Visceral and Thoracic Surgery, University Medical Centre Hamburg-Eppendorf, 20246 Hamburg, Germany
[15]Immunology and Allergy Unit, Department of Medicine Solna, Karolinska Institute, 17177 Solna, Sweden
[16]Martin Zeitz Centre for Rare Diseases, University Medical Centre Hamburg-Eppendorf, 20246 Hamburg, Germany
[17]Present address: Department of Microbiology and Immunology, The University of Melbourne at The Peter Doherty Institute for Infection and Immunity, Melbourne, VIC 3000, Australia
[18]These authors contributed equally
[19]Senior author
[20]Lead contact
*Correspondence: c.schramm@uke.de
https://doi.org/10.1016/j.xcrm.2024.101620

SUMMARY

Primary sclerosing cholangitis (PSC) is an immune-mediated liver disease of unknown pathogenesis, with a high risk to develop cirrhosis and malignancies. Functional dysregulation of T cells and association with genetic polymorphisms in T cell-related genes were previously reported for PSC. Here, we genotyped a representative PSC cohort for several disease-associated risk loci and identified *rs56258221* (*BACH2*/*MIR4464*) to correlate with not only the peripheral blood T cell immunophenotype but also the functional capacities of naive CD4+ T (CD4+ T_N) cells in people with PSC. Mechanistically, *rs56258221* leads to an increased expression of miR4464, in turn causing attenuated translation of BACH2, a major gatekeeper of T cell quiescence. Thereby, the fate of CD4+ T_N is skewed toward polarization into pro-inflammatory subsets. Clinically, people with PSC carrying *rs56258221* show signs of accelerated disease progression. The data presented here highlight the importance of assigning functional outcomes to disease-associated genetic polymorphisms as potential drivers of diseases.

INTRODUCTION

Primary sclerosing cholangitis (PSC) is a progressive inflammatory liver disease leading to obliteration of the intra- and/or extrahepatic bile ducts. Due to the widely unknown pathogenesis of PSC, there is no effective pharmacological therapy with a proven impact on the course of disease, and patients frequently develop end-stage liver disease and require liver transplantation.[1,2]

One presumable key contributor to the likely multifactorial disease pathogenesis is a dysregulated T cell function, including both CD4+ and CD8+ T cells.[3–5] We previously

© 2024 The Authors. Published by Elsevier Inc.
This is an open access article under the CC BY license (http://creativecommons.org/licenses/by/4.0/).

described increased frequencies of CD4$^+$ T helper (T_H) 17 cells and decreased frequencies of regulatory T cells (T_{REG}) within the peripheral blood of people with PSC compared to healthy donors and people with other inflammatory liver diseases.[6,7] Moreover, we have recently shown that CD4$^+$ T cells with a naive-like phenotype, showing a high capacity to polarize toward T_H17 cells, are increased in the blood and livers of people with PSC.[8] This is in line with the observation of an increased T_H17 response upon pathogen encounter by PSC-derived T cells *in vitro*,[9] supporting the hypothesis that T cells in PSC are prone to acquire a pro-inflammatory phenotype.[1,2]

However, the mechanisms causing this misguided immune cell differentiation are poorly understood. Besides environmental factors such as microbiota composition in intestine and bile ducts, genetic predisposition might contribute to T cell phenotype.[10,11] Previously, multiple genome-wide association studies have associated PSC to gene polymorphisms related to immune cell and particularly T cell function (e.g., *IL2RA*, *BACH2*, *FOXP1*, and *CD28*).[12–17] Of note, most of the PSC-related polymorphisms are also associated with other autoimmune diseases, e.g., type 1 diabetes or rheumatoid arthritis, and only few studies have so far reported on the functional implications of these polymorphisms for T cells, which is particularly true for PSC.[1,7,18]

We hypothesized that genetic predisposition contributes to T cell dysregulation in PSC and investigated the functional role of selected T cell-related gene polymorphisms (*CD28/CTLA4*, *IL2RA*, *FOXP1*, and *BACH2*) on the fate and function of T cells. Particularly, BACH2 (BTB domain and CNC homolog 2) has been shown to be a critical transcription factor for differentiation and maturation of both T and B lymphocytes. BACH2, initially identified as a transcriptional repressor in B cells,[19] has emerged as a central regulator also of T cells, with its targets including central transcription factors of T cell activation, e.g., *PRDM1* and *RUNX3*.[20–22] Knockout studies in mice have shown that lack of BACH2 leads to spontaneous T cell differentiation toward effector T cells as well as reduced numbers of T_{REG}.[23] In contrast, its overexpression was shown to promote a less differentiated "stem-like" T cell phenotype,[24] underlining its potential role as a gatekeeper of quiescence in naive T cells. Of note, the SNP *rs56258221* is located in the intergenic region between *BACH2* and *MIR4464*, an miRNA previously imputed to target BACH2 3′ untranslated region (UTR).[25]

We here identified this intergenic SNP, which was present in approximately 21% of people with PSC and 18% of controls in the study identifying this risk locus,[16] to associate with a distinct peripheral blood immunophenotype and skewed polarization of naive CD4$^+$ T (CD4$^+$ T_N) cells toward inflammatory T_H cell subsets. Functionally, this polymorphism enhanced expression of miR4464 and thereby led to reduced BACH2 protein levels in CD4$^+$ T_N, confirming the regulatory role of miR4464 on *BACH2* translation.

Overall, these data reveal a potential mechanism by which genetic predisposition shapes T cell function in autoimmunity and identify miRNAs and BACH2 as potentially druggable targets for the treatment of PSC.

RESULTS

People with PSC carrying the risk variant *rs56258221* show a distinct T cell phenotype compared to non-carriers

Peripheral blood samples were collected from people with PSC, and comprehensive immunophenotyping via flow cytometry yielded extensive T cell phenotypes of a representative PSC cohort (N = 36). The cohort was then genotyped for the PSC-associated polymorphisms *rs56258221* (*BACH2/MIR4464*), *rs80060485* (*FOXP1*), *rs4147359* (*IL2RA*), and *rs7426056* (*CD28/CTLA4*)[16] (Figure 1A). We identified *rs56258221* genotypes within the PSC cohort to separate our dataset into two cohorts with distinct immunophenotypes overall (p = 0.002) (Figure 1B), which was not observed for the other SNPs investigated (Figures S1A–S1C). Within this dataset, we detected several subsets of both CD4$^+$ and CD8$^+$ T cells to significantly differ in frequency between SNP carriers and non-carriers (Figures 1B and 1C). CD4$^+$ T_N (Figure 1D, p = 0.034) and T_H17 cells (Figure 1E, p = 0.036; Figure 1F, p = 0.038) were among the subsets with increased frequencies in SNP carriers,[26,27] whereas the frequency of T_{REG} was comparable between the two groups (Figure 1G, p = 0.355; Figure 1H, p = 0.489). Similar to CD4$^+$, CD8$^+$ T cells showed a trend toward increased T_N (Figure 1I, p = 0.064) but not for terminally differentiated T cells re-expressing CD45RA (T_{EMRA}) (Figure 1J, p = 0.325), which had previously been associated with changes in BACH2 levels in CD8$^+$ T cells.[21,24] However, we observed an increased frequency of CD39-expressing CD8$^+$ T cells, which points toward activated cells[28] in SNP carriers compared to non-carriers (Figure 1K, p = 0.003). In line with this, we observed a higher expression of pro-inflammatory cytokine tumor necrosis factor alpha (Figure 1L, p = 0.003) and granzyme B (GzmB) (Figure 1M, p = 0.054) in CD8$^+$ T cells of people with PSC carrying *rs56258221*. The expression of GzmB has previously been shown to be affected by BACH2.[20]

We next followed up on these observations by performing bulk RNA sequencing on liver tissue samples acquired via liver biopsies from people with PSC (n = 12). Notably, in carriers of *rs56258221*, we found significantly increased expression of C-C chemokine receptor type 6 (Figure S1N, p = 0.034) and killer lectin-like receptor B1 (Figure S1O, p = 0.018), which are commonly used markers to identify T cells that are polarized toward a T_H17-like phenotype.[29] Most other markers assessed, including genes associated with regulatory and activated phenotype of CD8$^+$ T cells, were not changed between SNP carriers and non-carriers (Figures S1P–S1U).

To generalize our findings about *rs56258221* affecting immune cell frequencies in the peripheral blood of people with PSC, we additionally performed immunophenotyping in cohorts of healthy blood donors (HD, n = 18), people with primary biliary cholangitis (PBC, n = 16), people with inflammatory bowel disease without PSC (IBD, n = 12), and people with metabolic dysfunction-associated steatohepatitis (MASH, n = 6). However, we did not observe any significant differences in the populations mentioned previously between carriers and non-carriers of *rs56258221* (Figures S1D–S1M).

CellPress
OPEN ACCESS

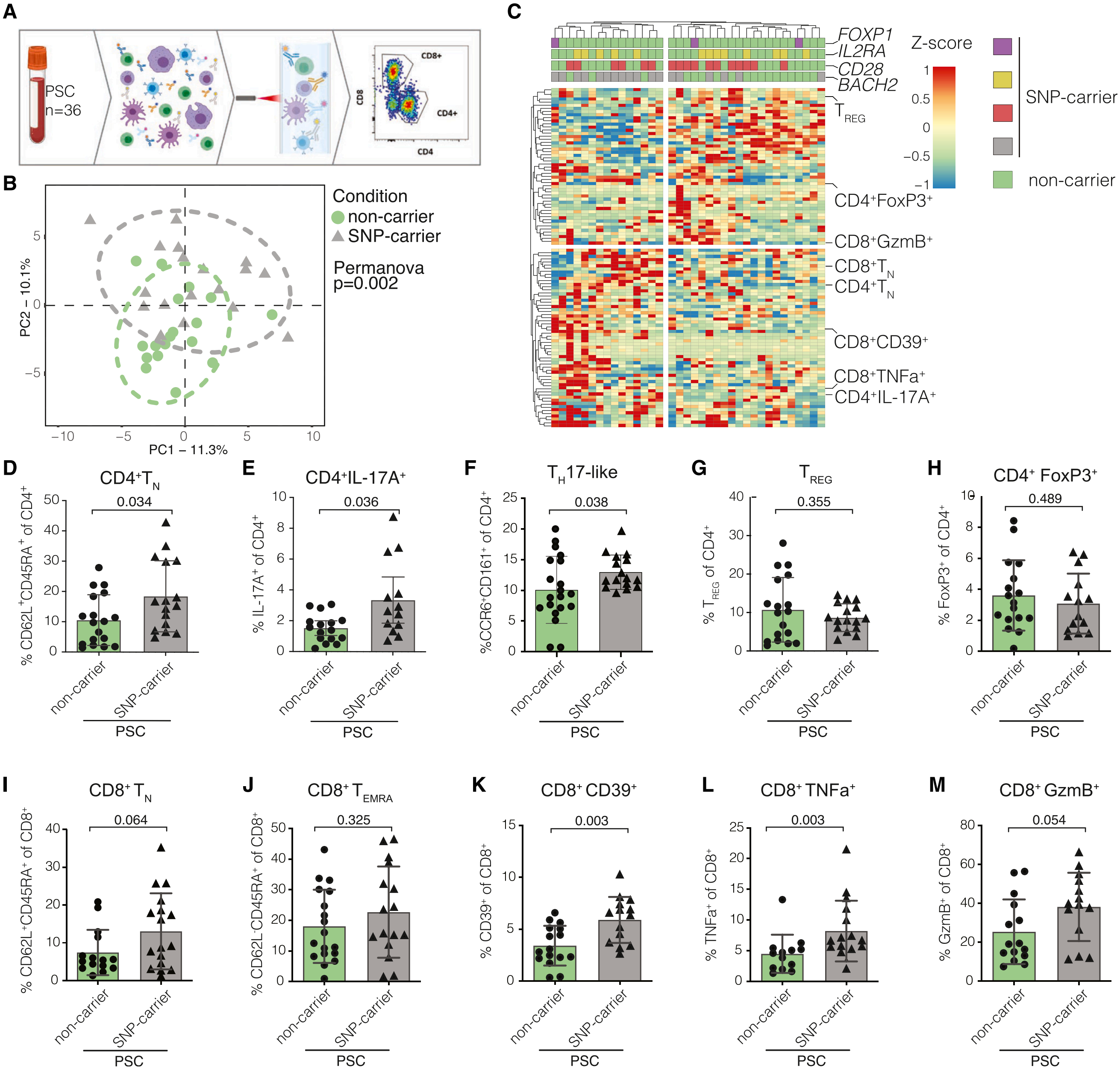

Figure 1. Risk variant *rs56258221* (BACH2/MIR4464) entails phenotypic differences in peripheral T cells in people with PSC

(A) Schematic depiction of the workflow for immunophenotyping.

(B) Principal component analysis of the analyzed immunophenotyping data, separated by the genotype for polymorphism *rs56258221*.

(C) Heatmap illustrating the immunophenotyping dataset ($n = 36$) and highlighting populations that differed in frequency between carriers of *rs56258221* ($n = 18$) and non-carriers ($n = 18$).

(D–H) Frequencies of different $CD4^+$ T cell subsets. (D) T_N, identified by CD62L/CD45RA. (E) T_H17 cells identified by IL-17A expression upon stimulation with PMA (phorbol 12-myristate 13-acetate)/ionomycin. (F) T_H17 cells identified by CCR6/CD161. (G) T_{REG} identified by CD127/CD25. (H) T_{REG} identified by FoxP3 expression upon stimulation with PMA/ionomycin.

(I–M) Frequencies of different $CD8^+$ T cell subsets. (I) T_N, identified by CD62L/CD45RA. (J) T_{EMRA}, identified by CD62L/CD45RA. (K) CD39 expression on $CD8^+$ T cells. (L) TNF expression upon stimulation with PMA/ionomycin. (M) GzmB expression upon stimulation with PMA/ionomycin. Characteristics of the clinical cohort are included in Table S1. Statistics: normality distribution was tested by Kolmogorov-Smirnov test; normal distribution: Welch's t test; no normal distribution: Mann-Whitney U test. $p < 0.05$ was considered statistically significant. Data are presented as mean ± SD and deriving from $n \geq 2$ repeats per experiment.

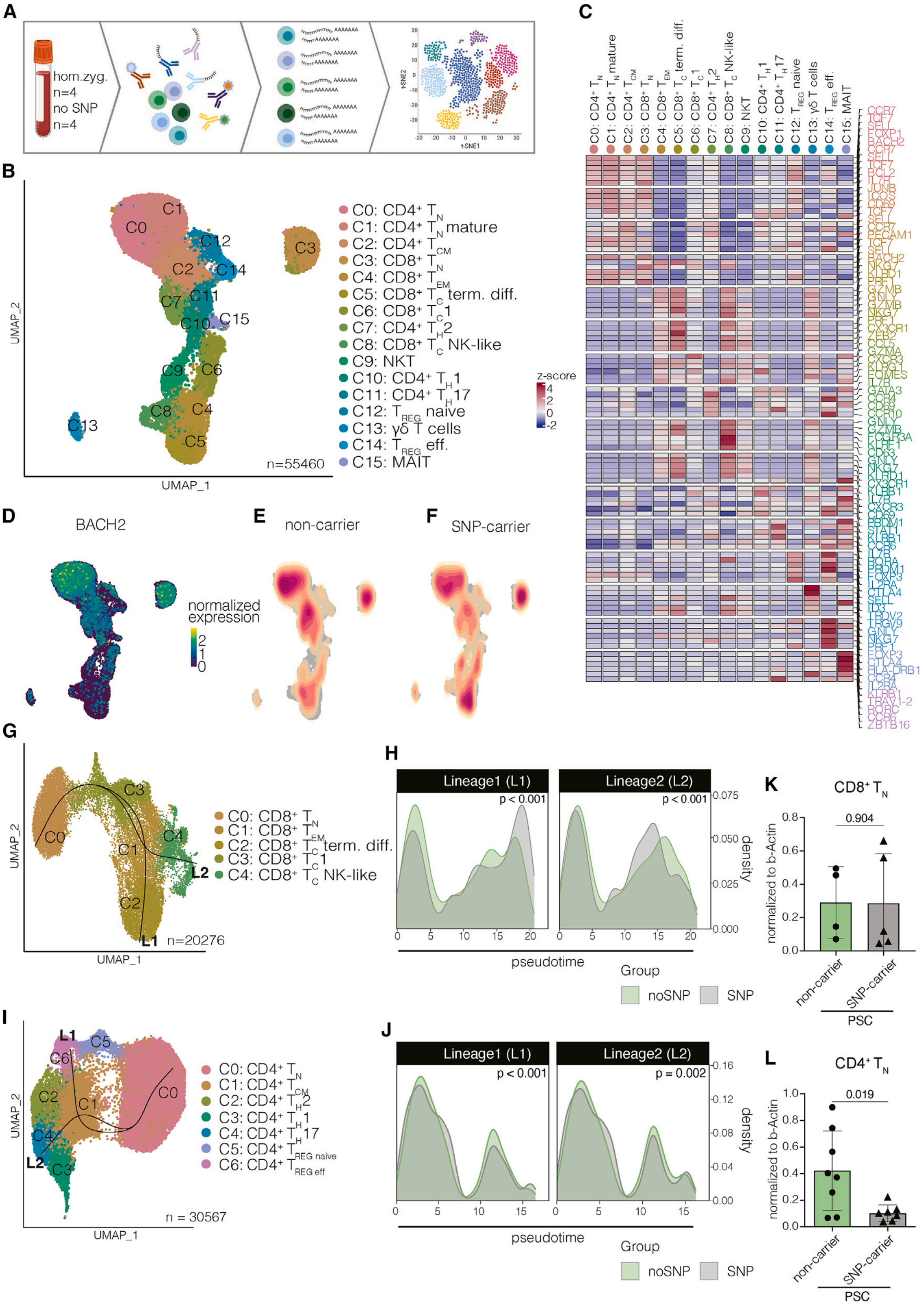

(legend on next page)

Developmental trajectories of peripheral blood CD4$^+$ and CD8$^+$ T_N are altered in carriers of *rs56258221*

In order to investigate whether carrying *rs56258221* affects the overall transcriptome of T cells and thereby shapes their cellular state, we performed cellular indexing of transcriptomes and epitopes by sequencing (CITE-Seq) on peripheral blood-derived T cells from people with PSC either homozygous for *rs56258221* (n = 4) or non-carriers (n = 4). We sequenced 55,460 T cells across eight individuals and identified most of the major CD4$^+$ (T_N, central memory [T_{CM}], T_H1, T_H2, T_H17, and T_{REG}) and CD8$^+$ T cell subsets (T_N, T_{CM}, effector memory [T_{EM}], cytotoxic [T_C]), as well as gamma delta T cells (gd T cells), mucosa-associated invariant T cells (MAIT), and natural killer T cells (NKT) via analysis of differentially expressed genes combined with surface protein expression (Figures 2A–2C and S2A). After confirming T_N as the major source of *BACH2* expression among these clusters (Figure 2D), we compared *BACH2* expression levels between carriers of *rs56258221* and non-carriers but observed no differences between the groups (Figure S2B). We next compared the cell composition between the two genotypes among the clusters and noted a dissimilar distribution of cells within the CD4$^+$ T_N cluster as well as the CD8$^+$ T_C terminally differentiated cluster (Figures 2E and 2F). Hypothesizing on the effects of carrying *rs56258221* on differentiation of T_N, we excluded NKT, MAIT, and gd T cells and split the dataset into CD4$^+$ and CD8$^+$ T cells. We then utilized *slingshot* combined with *condiments*[30,31] to compare the differentiation capacities between carriers of *rs56258221* and non-carriers. Interestingly, we detected significant differences in cellular distribution along the trajectories of both CD4$^+$ and CD8$^+$ T cells (Figures 2G–2J).

Considering these findings and also previously published studies on the effect of altered BACH2 levels on T cell differentiation and phenotype,[21–24,26] we quantified BACH2 protein levels in fluorescence-activated cell-sorted peripheral blood CD4$^+$ and CD8$^+$ T_N. Notably, we detected a strong reduction of BACH2 protein levels in CD4$^+$, but not CD8$^+$ T_N (Figure 2K–2L, p = 0.019 and p = 0.904) in homozygous carriers of *rs56258221*. Combined, we observed phenotypic differences in peripheral blood T cells between the two genotypes. These differences were accompanied by reduced BACH2 protein levels in CD4$^+$ but not CD8$^+$ T_N, indicating a possible direct effect of *rs56258221* on BACH2 expression in CD4$^+$ T_N.

CD4$^+$ T_N cells from carriers of *rs56258221* show a developmental propensity toward a pro-inflammatory phenotype *in vitro*

Based on the phenotypical changes in CD4$^+$ T cells and the reduced BACH2 protein levels in CD4$^+$, but not CD8$^+$ T_N of SNP carriers, we followed up on this by re-clustering the CD4$^+$ T_N clusters from our CITE-Seq dataset in order to investigate potential differences between the genotypes. Re-clustering resulted in four subclusters of CD4$^+$ T_N, which we annotated according to their gene expression profiles (SC0_resting state, SC1_activated state, SC2_proliferating state, and SC3_non-resting state) (Figures 3A and 3B). The expression levels of *BACH2* mRNA among the subclusters were similar between cells from carriers of *rs56258221* and non-carriers (Figure 3C). Interestingly, though, the clusters assigned to activated transcriptomes (SC1 and SC2) showed a trend toward enrichment in SNP carriers (Figure 3D).

Next, we decided to perform functional experiments on CD4$^+$ T_N *in vitro* to assess potential differences in response to stimulation (Figure 3E).

We previously demonstrated that CD4$^+$ T_N cells from people with PSC show increased T_H17 polarization capacities *in vitro*.[6,8] We observed that CD4$^+$ T_N cells from people with PSC show a higher propensity to polarize not only toward T_H17 cells (Figure 3F, p = 0.007) but also toward T_H1 cells, compared to controls (Figure 3G, p = 0.040). Notably, the propensity of PSC-derived CD4$^+$ T_N cells to polarize toward pro-inflammatory subsets was accompanied by a significantly lower rate of polarization toward induced T_{REG} (iT_{REG}) (Figure 3H, p = 0.001). These observations suggest a shift in the developmental propensities of CD4$^+$ T_N cells in PSC toward a pro-inflammatory phenotype, in line with the previously reported imbalance of T_H17 and T_{REG} frequencies in peripheral blood of people with PSC and the propensity of liver-resident naive-like CD4$^+$ T cells to differentiate into T_H17 cells.[7–9] We next assessed whether genetic predisposition contributes to the observed differences by comparing carriers and non-carriers of the PSC-associated genetic polymorphisms assessed in this study (*CD28/CTLA4*, *IL2RA*, *FOXP1*, and *BACH2/MIR4464*). In line with the previous results, we observed an association of *rs56258221* (*BACH2/MIR4464*) with a pro-inflammatory polarization of CD4$^+$ T_N, which was particularly true for the T_H17/iT_{REG} dichotomy (Figures 3I–3K; T_H17 p = 0.041, iT_{REG} p = 0.044). This association was not present for the other

Figure 2. Developmental trajectories of peripheral blood T_N are altered in carriers of *rs56258221*

(A) Schematic depiction of the workflow for CITE-Seq (n = 4 homozygous carrier; n = 4 non-carrier) (all 8 patients cisgender males).

(B) Uniform manifold approximation and projection (UMAP) resembling 55,460 peripheral blood T cells from people with PSC either homozygous for or not carrying *rs56258221*, subdivided into 16 clusters by Seurat.

(C) Heatmap highlighting differentially expressed genes used to assign cell types to clusters.

(D) Expression of BACH2 mapped onto the UMAP from (B), highlighting T_N as the main population expressing BACH2.

(E and F) Cellular density within landscape of peripheral blood T cells from (J), separated into SNP carriers (E) and non-carriers (F).

(G) Subclustering of CD8$^+$ T cells and trajectory analysis via *slingshot* with CD8$^+$ T_N as the selected starting point.

(H) Differential progression analysis via *condiments* to identify differential distribution of cells along trajectory.

(I) Subclustering of CD4$^+$ T cells and trajectory analysis via *slingshot* with CD4$^+$ T_N as the selected starting point.

(J) Differential progression analysis via *condiments* to identify differential distribution of cells along trajectory.

(K) Relative BACH2 expression compared to B-actin expression via western blot from FACS-sorted CD8$^+$ T_N (n = 9).

(L) Relative BACH2 expression compared to B-actin expression via western blot from FACS-sorted CD4$^+$ T_N (n = 15). Characteristics of the clinical cohort are included in Tables S2 and S3. Statistics: normality distribution was tested by Kolmogorov-Smirnov test; normal distribution: Welch's t test; no normal distribution: Mann-Whitney U test. $p < 0.05$ was considered statistically significant. Data are presented as mean ± SD and deriving from $n \geq 2$ repeats per experiment.

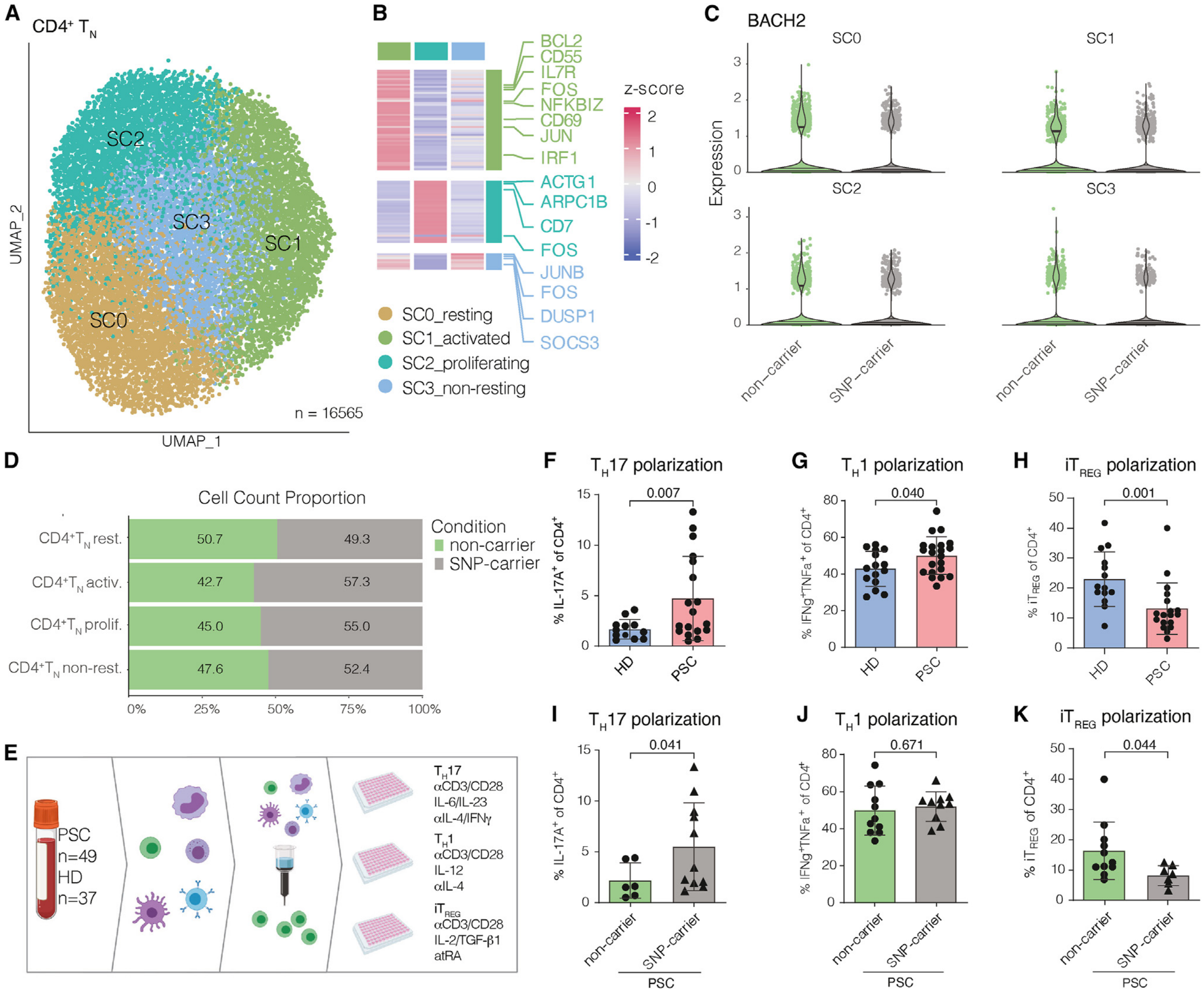

Figure 3. PSC-associated polymorphism *rs56258221* (*BACH2/MIR4464*) contributes to increased polarization of CD4+ T_N cells toward pro-inflammatory phenotypes

(A and B) Re-clustering of CITE-Seq data from CD4+ T_N (cluster C0 from Figure 1J) resulted in four subclusters of CD4+ T_N (SC0-3) (A), which were assigned to cellular states via analysis of differentially expressed genes (B). SC0 did not show differentially expressed genes.

(C) Expression levels of *BACH2* among each cluster.

(D) Bar plot comparing the frequencies of SC0-3 between SNP carriers and non-carriers.

(E) Schematic depiction of the workflow for *in vitro* polarization assays.

(F) After 12 days of polarizing culture of CD4+ T_N from people with PSC (n = 18) and HD (n = 12), the frequency of CD4+IL-17A+ cells was determined.

(G) After 7 days of polarizing culture of CD4+ T_N from people with PSC (n = 21) and HD (n = 15), the frequency of T CD4+CXCR3+TBET+IFNg+TNFa+ cells was determined.

(H) After 7 days of polarizing culture of CD4+ T_N from people with PSC (n = 18) and HD (n = 14), the frequencies of CD4+CD25+CD152+FOXP3+ cells were determined.

(I–K) Data on people with PSC from T_H17 (F), T_H1 (G), and iT_{REG} (H) polarization were separated by genotype for polymorphism *rs56258221*. Characteristics of the clinical cohort are included in Tables S4–S6. Statistics: normality distribution was tested by Kolmogorov-Smirnov test; normal distribution: Welch's t test; no normal distribution: Mann-Whitney U test. $p < 0.05$ was considered statistically significant. Data are presented as mean ± SD and deriving from $n \geq 2$ repeats per experiment.

SNPs investigated (Figures S3A–S3H) and was not linked to differences in proliferation of CD4+ T_N (Figures S3I–S3K).

Taken together, our findings on the polymorphism *rs56258221* (*BACH2/MIR4464*) support the hypothesis of genetic predisposition contributing to skewed CD4+ T_N polarization capacities in PSC.

The risk variant *rs56258221* is associated with increased expression of miR4464 in CD4+ T_N

To assess whether epigenetic changes are linked to the observed differences in phenotype and differentiation, we utilized our previously published dataset on single-cell sequencing

CellPress
OPEN ACCESS

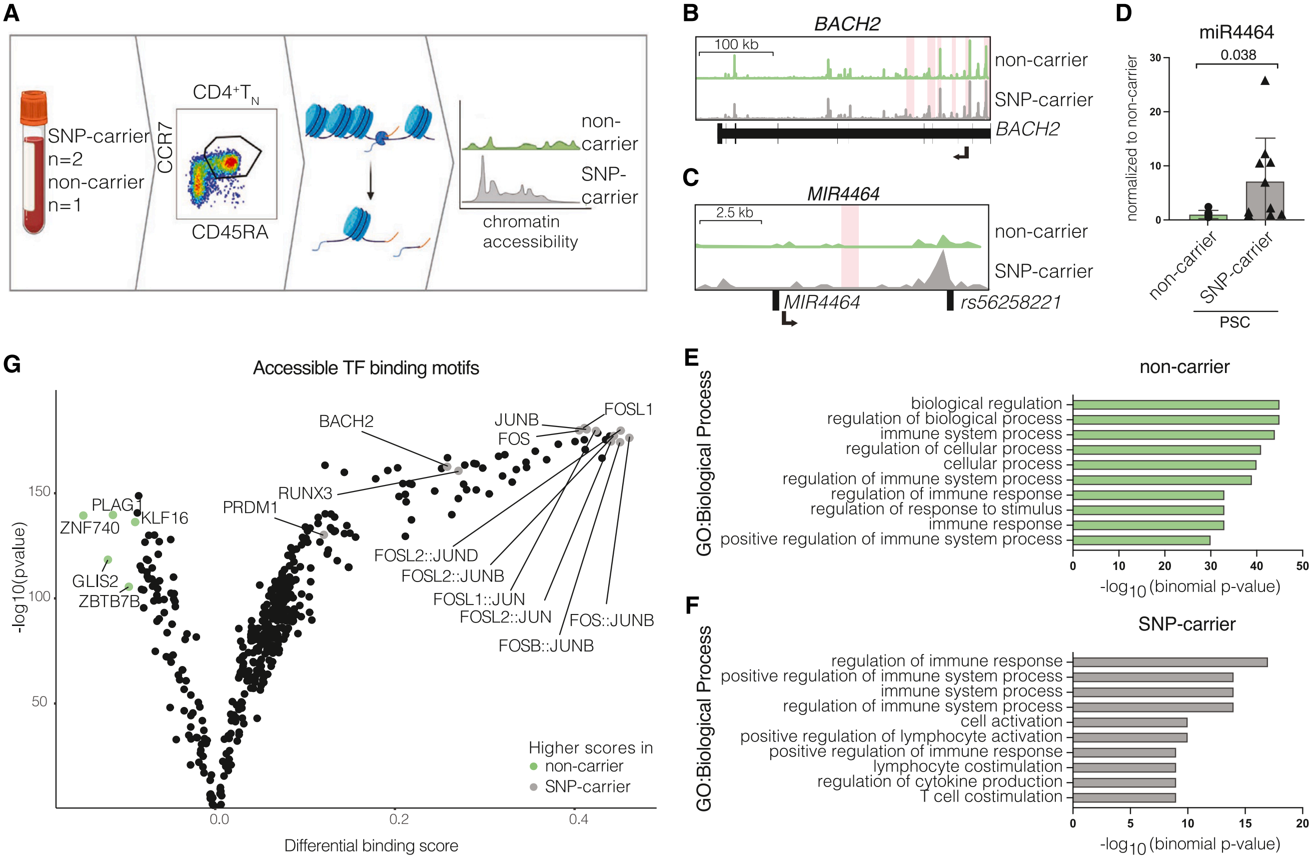

Figure 4. The risk variant *rs56258221* (*BACH2/MIR4464*) is associated with lower BACH2 protein levels and increased expression of miR4464

(A) Schematic depiction of the workflow for scATAC-Seq analysis (n = 4).

(B) Coverage plot showing the chromatin region of *BACH2*. Significantly increased accessible chromatin regions in SNP carriers (n = 2) are highlighted in red.

(C) Coverage plot showing the chromatin region surrounding *rs56258221*, including the *MIR4464* locus. Significantly increased accessible chromatin regions in SNP carriers (n = 2) are highlighted in red.

(D) Detection of miR4464 in FACS-sorted CD4$^+$ T$_N$ of homozygous carriers and non-carriers of *rs56258221*.

(E and F) Gene ontology (GO) terms representing total accessible chromatin regions from either SNP carriers (E) or non-carriers (F). The ten GO terms with lowest p values are displayed.

(G) Assignment of transcription factor-binding sites (TFBS) to accessible chromatin regions via *TOBIAS*. Analysis of differential TFBS accessibility between carriers of *rs56258221* (n = 2) and non-carriers (n = 2) is shown. Characteristics of the clinical cohort are included in Table S3. Statistics: normality distribution was tested by Kolmogorov-Smirnov test; normal distribution: Welch's t test; no normal distribution: Mann-Whitney U test. p < 0.05 was considered statistically significant. Data are presented as mean ± SD and deriving from $n \geq 2$ repeats per experiment.

assay for transposase-accessible chromatin (scATAC-Seq) on CD4$^+$ T$_N$ cells from people with PSC[8] and retrospectively identified SNP carriers (n = 2) and non-carriers (n = 1) within this dataset (Figure 4A). We detected the *BACH2* locus to be more accessible in CD4$^+$ T$_N$ from carriers of *rs56258221* (Figure 4B), which was unexpected considering the reduction of BACH2 protein. As the intergenic polymorphism *rs56258221* is assigned to both *BACH2* and *MIR4464*, we hypothesized on post-transcriptional regulation contributing to the observed reduction in BACH2 protein.

Of note, miR4464 was previously imputed to exert regulatory function on BACH2, as its binding motifs are located within the 3′ UTR of the BACH2 mRNA.[25]

To address our hypothesis on miR4464 being involved in the observed reduction of BACH2 protein levels, we went back to the scATAC-Seq dataset and observed a pronounced increase of chromatin accessibility spanning the genomic location of *rs56258221* (*Chr6:90,320,722*), which is in close proximity to *MIR4464* (Figure 4C). Next, we used fluorescence-activated cell-sorted CD4$^+$ T$_N$ from the same people with PSC included in our single-cell RNA sequencing and western blot experiments to determine expression levels of miR4464. Notably, we observed a significantly higher expression of miR4464 in CD4$^+$ T$_N$ from carriers of *rs56258221* compared to non-carriers (Figure 4D, p = 0.038).

In addition, gene ontology analysis of overall accessible chromatin sites suggested an activated cellular phenotype for CD4$^+$ T$_N$ from carriers of *rs56258221*, indicated by the terms "cell activation," "positive regulation of lymphocyte activation," and "T cell costimulation" (Figure 4E). In contrast, CD4$^+$ T$_N$ cells

from non-carriers were linked to a presumably steady cellular state (Figure 4F). To analyze this finding in more detail, we assigned transcription factor-binding sites to accessible chromatin regions by utilization of *TOBIAS* (Transcription factor Occupancy prediction By Investigation of ATAC-seq Signal).[32] We observed an increase in accessible binding sites of transcription factors associated with T cell activation, particularly members of the AP-1 complex (BATF, FOS, JUN, and JDP), Blimp-1 (PRDM1), and Runx3 (RUNX3), which are all known targets of BACH2,[20,21] in carriers of *rs56258221* (Figure 4G). In line with this, $CD4^+$ T cells from SNP carriers also showed an increase in BACH2-binding sites, indicating less binding activity of BACH2 as a transcriptional repressor (Figure 4G).

Combined, we show reduced protein levels of the transcriptional repressor BACH2 accompanied by increased expression of miR4464 in carriers of the risk variant *rs56258221*. This reduction is associated to an increase of accessible motifs of both BACH2 and its targets in the epigenetic landscape of $CD4^+$ T_N.

Increased expression of miR4464 attenuates BACH2 translation in $CD4^+$ T_N, resulting in a reduced polarization capacity toward iT_{REG}

After identifying increased expression of BACH2-targeting miR4464 as a potential cause for the observed reduction of BACH2 protein in $CD4^+$ T_N, we investigated whether miR4464 functionally contributes to this effect by downregulating the translation of *BACH2* mRNA in carriers of *rs56258221*. Therefore, we isolated $CD4^+$ T_N from peripheral blood of healthy donors (n = 12) and transfected the cells with miR4464. Next, we performed polarizing *in vitro* culture toward iT_{REG}, using the same experimental setup as previously described (Figure 5A). First, we controlled for successful transfection by determining the level of miR4464, which showed an approximate 10-fold increase compared to cells transfected with non-binding miRNA (mock) and only slightly decreased during 72 h post transfection (Figure 5B). Of note, the observed increase was in a similar range to the changes observed between homozygous carriers of *rs56258221* and non-carriers (cf. Figure 4D). Next, we determined the expression levels of both *BACH2* mRNA and protein and observed no profound effect of the transfection with miR4464 on *BACH2* mRNA but a significant reduction of BACH2 protein (Figures 5C and 5D, p = 0.028). We next cultured transfected $CD4^+$ T_N under iT_{REG}-polarizing conditions. Notably, we observed a significant decrease in iT_{REG} formation in healthy non-carriers of *rs56258221* upon transfection with miR4464, compared to mock-transfection, which was not seen for SNP carriers (Figure 5E, p = 0.026). This observation is potentially due to the increased miR4464 levels per se in SNP carriers, which we described in the section earlier. To finally prove that an increase in miR4464 directly impacts *BACH2* translation, we cloned the fragment from the 3′ UTR region of *BACH2* mRNA containing the predicted miR4464-binding site[25] or a mutated binding site into the *pmirGLO dual-luciferase vector*. Transfection of HEK293T cells with the *BACH2* tester plasmid in combination with miR4464 resulted in strongly reduced luciferase signals compared to mock transfection, pointing toward an effective targeting of the cloned 3′ UTR region of *BACH2* mRNA by miR4464 (Figure 5G, p = 0.004). Importantly, this effect was not observed upon transfection with plasmids containing a mutated miR4464 binding site within the 3′ UTR region of *BACH2* (Figure 5H, p = 0.333), confirming the imputed data that were previously reported on miR4464 targeting *BACH2* mRNA.[25]

Combined, our data show that miR4464 indeed binds the 3′ UTR region of *BACH2* mRNA and thereby directly regulates protein translation of this central transcriptional repressor, which had an immediate impact on the capability of $CD4^+$ T_N to polarize into iT_{REG}.

Markers of disease severity in people with PSC carrying *rs56258221*

To investigate whether the previously shown differences in T cell differentiation and phenotype associate with variations in the clinical course of people with PSC, we evaluated a selection of routine laboratory parameters of people with PSC attending the outpatient clinic for gastroenterology and hepatology at the University Medical Centre Hamburg-Eppendorf (n = 210). Using SNP genotyping, we identified 74 carriers and 136 non-carriers of *rs56258221* in our cohort. Both groups showed similar age at diagnosis (36.1 vs. 35.8 years) and duration of disease at the time of data collection (12.6 vs. 12.3 years) (Figure 6A).

Intriguingly, biomarkers routinely used to evaluate severity of disease differed between carriers and non-carriers of *rs56258221*. These included increased levels of aspartate aminotransferase (AST, 69 vs. 48 U/L, p = 0.007), alanine aminotransferase (ALT, 72 vs. 53 U/L, p = 0.035), and total bilirubin (2.9 vs. 1.6 mg/dL, p = 0.047) (Figure 5A) in carriers of *rs56258221*. In addition, carriers showed a higher rate of liver transplantation (LTx), although this difference was not statistically significant (21.6% vs. 13.2%, p = 0.120).

Consequently, we aimed to further validate a potential effect of *rs56258221* on the clinical course of people with PSC. Therefore, we extended our PSC cohort from Hamburg (n = 210) with an independent PSC cohort (n = 137) from the Norwegian PSC Center at the *Oslo University Hospital*, *Rikshospitalet* that included 100 people with PSC who had received LTx. In the analysis of a 15-year period post diagnosis in 347 people with PSC, there was a trend of carriers (n = 132) toward a more severe clinical course compared to non-carriers (n = 215) of *rs56258221* (log rank test: p = 0.069) (Figure 6B).

DISCUSSION

Both genetic and environmental factors contribute to the pathogenesis of PSC, a dreadful liver disease lacking any effective pharmacological treatment option. In most people with PSC, chronic disease progression inevitably leads to end-stage liver disease, death, or liver transplantation within two decades.[1,10] Despite the fact that over 20 gene polymorphisms have been assigned to elevated risk for PSC, data on functional consequences of these polymorphisms are scarce,[7,18] which is most likely due to their intronic and intergenic locations.[1,12–17,33] We here hypothesized that PSC-associated genetic variants contribute to the dysregulation of T cells we and others have described previously in people with PSC.[6–9,18,34]

The majority of the PSC-associated polymorphisms is located within or nearby genetic loci related to T cell function. Among

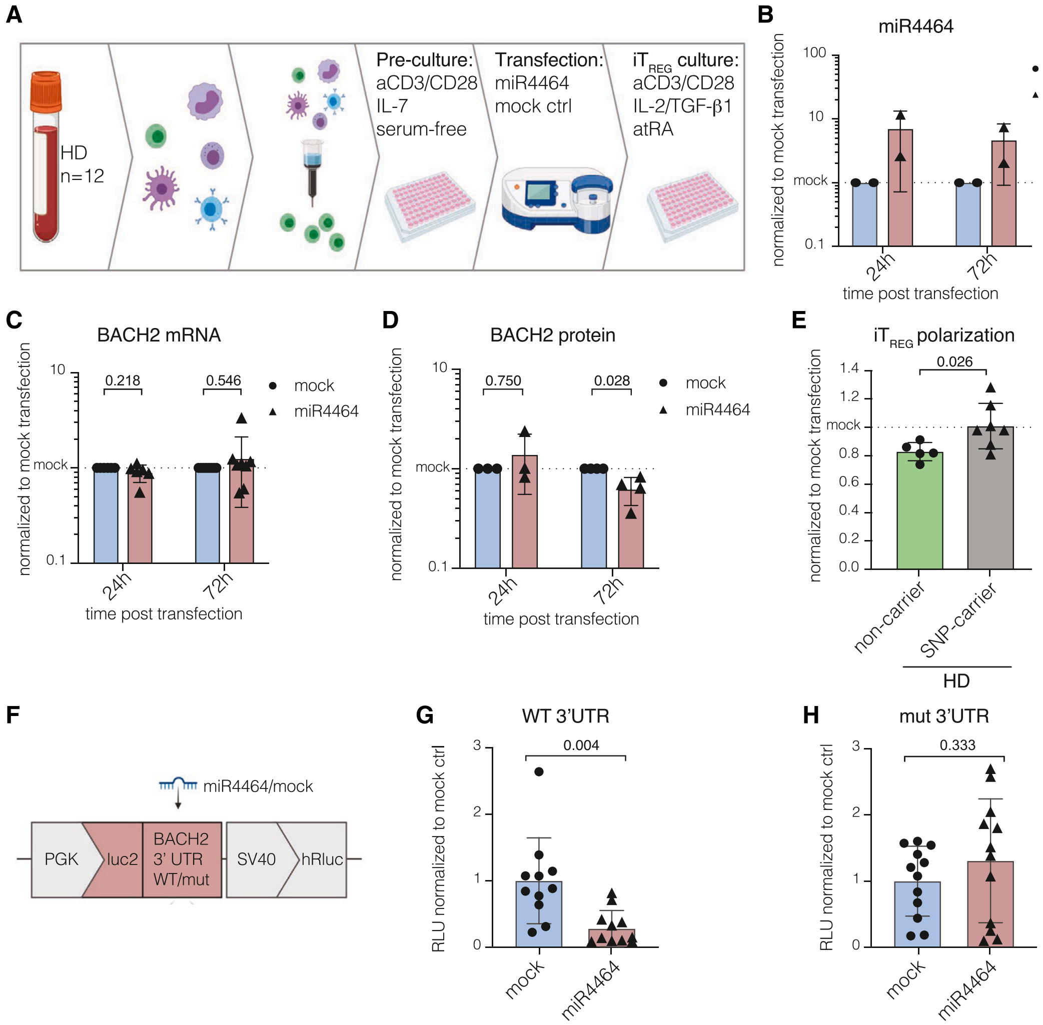

Figure 5. miR4464 induces reduced BACH2 protein translation in CD4^{+} T_N and reduces polarization capacity toward iT_{REG}

(A) Schematic depiction of the workflow for the *in vitro* transfection of CD4^{+} T_N with miR4464.

(B) Detection of miR4464 levels in CD4^{+} T_N cells 24 and 72 h post transfection.

(C) Detection of BACH2 mRNA levels in CD4^{+} T_N cells 24 and 72 h post transfection.

(D) Quantified western blot analysis on BACH2 protein levels from CD4^{+} T_N cells 24 and 72 h post transfection.

(E) *In vitro* polarization toward iT_{REG}. Frequencies of iT_{REG} 6 days post transfection with miR4464. Data were normalized to the respective mock transfection controls.

(F) Schematic depiction of the experimental setup utilizing the pmirGLO tester plasmid containing the 3′ UTR of BACH2 mRNA or a mutated control site.

(G and H) Luciferase activity was determined from HEK293T cells 24 h post co-transfection of either miR4464 or mock control and the tester plasmid containing a mutated control binding site (G) or the wild-type binding site (H). Luciferase activity was normalized to include Renilla luciferase (hRluc) control. Statistics: normality distribution was tested by Kolmogorov-Smirnov test; normal distribution: Welch's t test; no normal distribution: Mann-Whitney U test. $p < 0.05$ was considered statistically significant. Data are presented as mean ± SD and deriving from $n \geq 2$ repeats per experiment.

these, we identified the intergenic polymorphism *rs56258221* at the *BACH2/MIR4464* gene locus to affect the phenotype of both CD4^{+} and CD8^{+} T cells and, in particular, the fate and function of CD4^{+} T_N. We demonstrate that CD4^{+} T_N cells from people with PSC display skewed polarization capabilities toward pro-inflammatory subsets, i.e., T_H1 and T_H17. In contrast, the polarization of CD4^{+} T_N toward anti-inflammatory iT_{REG} was impaired. This is of interest as T_H17 and its signature cytokine IL-17 are widely recognized as key players in PSC pathogenesis and autoimmunity.[1,2,6,8,9] Moreover, the presence of *rs56258221* characterized a population with increased frequencies of T_H17 cells, and SNP carriers showed a skewed *in vitro* differentiation of CD4^{+} T_N toward T_H17 and away from iT_{REG} cells.

BACH2 is a transcriptional repressor playing an essential role in T cell quiescence and activation, as well as in the maintenance of CD4^{+} T cell subsets, i.e., T_{REG}, T_H1, and T_H17 cells.[19–23,29] Although the exact mechanism of action of BACH2 remains to be determined, its interference with signaling of both T cell and IL-2 receptor has been described for murine T_{REG}.[23,35] Intriguingly, the transfer of BACH2-deficient T_{REG} into recipient mice

A

	SNP-carrier (n = 74)	non-carrier (n = 136)	p-value
Age at study (y)	52.8	52.4	
Sex (m/f)	54/20	79/57	
IBD frequence (%)	68	67	
Time between diagnosis and data acquisition (y)	12.6 ± 7.1	12.3 ± 8.9	n.s. (0.46)
LTX (%)	21.6	13.2	n.s. (0.12)
Age at diagnosis (y)	36.1 ± 11.2	35.9 ± 13.0	n.s. (0.88)
Transient elastography (kPa)	13.2 ± 15.6	14.4 ± 18.3	n.s. (0.39)
ALP (U/l)	233.0 ± 187.5	216.8 ± 177.0	n.s. (0.50)
GGT (U/l)	208.7 ± 303.0	157.1 ± 210.0	n.s. (0.45)
Total Bilirubin (mg/dl)	2.9 ± 5.2	1.6 ± 2.2	0.048
ASAT (U/l)	68.9 ± 58.8	48.1 ± 36.6	0.008
ALAT (U/l)	71.5 ± 63.8	53.1 ± 49.1	0.036

B

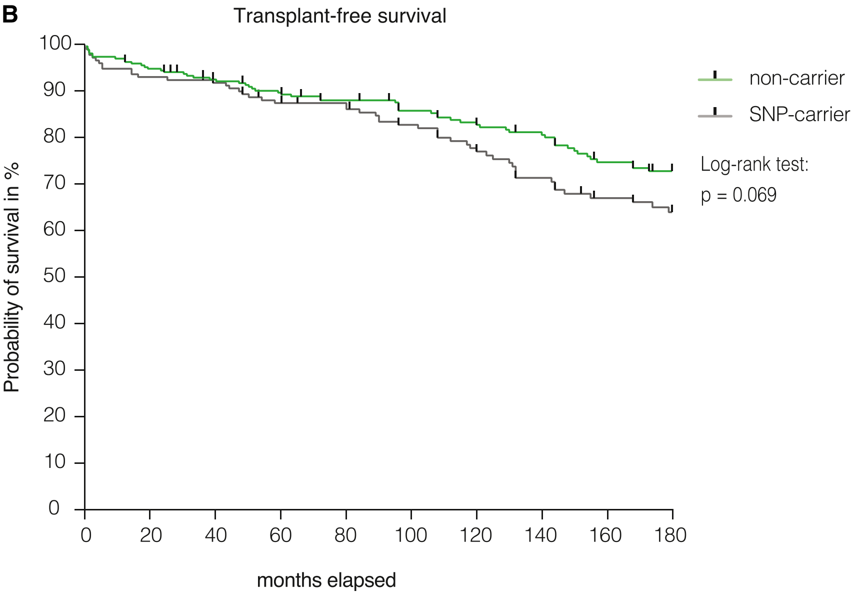

Figure 6. Clinical phenotype of people with PSC carrying polymorphism *rs56258221* (BACH2/MIR4464)

(A) Laboratory parameters and clinical features of people with PSC attending the outpatient clinic for gastroenterology and hepatology of the University Medical Centre Hamburg-Eppendorf (*n* = 210). The data were separated into carriers and non-carriers of *rs56258221*. Both groups span a similar time between diagnosis of PSC and collection of laboratory parameters (mean ± SD).

(B) Assessment of transplant-free survival between SNP carriers and non-carriers. The combined independent cohorts from University Medical Centre Hamburg-Eppendorf (Germany) and the Norwegian PSC Centre in Oslo (Norway) are shown. Survival data were analyzed by using log rank test. Tick marks represent censoring.

was shown to induce signs of autoimmunity.[35] However, so far, it was unknown whether and how the disease-associated SNP *rs56258221* influences the function of BACH2. Consequently, we analyzed mRNA and protein levels of BACH2 in CD4$^+$ T$_N$ isolated from people with PSC carrying and not carrying the risk allele *rs56258221* and found similar mRNA expression, but significantly lower BACH2 protein levels in SNP carriers.

In line with lowered BACH2 protein levels in CD4$^+$ T$_N$ from carriers of *rs56258221*, we observed more accessible BACH2-binding motifs in chromatin sequencing data, which represents its lower activity as a transcriptional repressor.[21,24] In addition to binding motifs of *BACH2* itself, we detected more accessible motifs of *PRDM1*, *RUNX3*, and members of the AP-1 transcriptional complex (e.g., Jun and Fos), all of which are known targets of BACH2[21,24,26] and involved in T$_H$17 cell formation and the development of T cell effector function.[36,37] Overall, chromatin accessibility suggested a more activated phenotype of CD4$^+$ T$_N$ in SNP carriers compared to non-carriers, associating lowered BACH2 protein levels to enhanced T cell activation and being in line with the observation of *BACH2*-deficient T cells spontaneously developing an activated phenotype.[26,38] Combined, these findings demonstrated an association of risk variant *rs56258221* with lower BACH2 protein levels and functional consequences in CD4$^+$ T cells. However, and in contrast to these findings, we observed a more accessible *BACH2* gene in SNP carriers compared to non-carriers. Hypothesizing on post-transcriptional regulation, we investigated the involvement of miR4464 and were able to link its increased expression and genetic accessibility to the reduced BACH2 protein levels in carriers of *rs56258221*. The *MIR4464* locus is located within the *cis*-regulatory region upstream of *BACH2*, and miR4464 has previously been suggested to target the 3′ UTR of *BACH2* mRNA using *in silico* analyses.[25] Finally, we were able to confirm the imputed regulation of *BACH2* translation through miR4464 by utilizing a luciferase-based plasmid system with mutated miR4464-binding sites.[39]

From a clinical perspective, our data suggest that *rs56258221* may contribute to disease progression and, if validated in independent studies, potentially enables risk stratification of people with PSC in the future. Considering the elevated risk for

hepatobiliary malignancies in people with PSC and the function of BACH2 in suppressing transcription factors of the AP-1 family, which are key molecules in tumorigenesis, our data should stimulate research into the effects of *rs56258221* in cholangiocytes.

Our study provides evidence for the previously imputed function of miR4464 in regulating BACH2 protein expression. Moreover, we link the autoimmunity-associated polymorphism *rs56258221* to dysregulation of CD4$^+$ T_N by shifting the balance of T_H17 and T_{REG} toward pro-inflammatory T_H17 subsets. In addition, CD8$^+$ T cells were found to be more activated in SNP carriers, but the mechanism was not linked to changes in BACH2 protein levels and remains to be elucidated. The data presented here should fuel more studies investigating the effects of disease-associated risk variants that, although mostly located in non-coding regions, may have an impact on cellular function and possibly on disease phenotype.

Limitations of the study

Limitations of our study include the fact that genetic polymorphisms occur in linkage disequilibrium, which complicates the annotation of a functional outcome to one specific polymorphism, especially when working with human material. Notably, *rs56258221* is in high linkage disequilibrium with *rs72928038*, which is an intronic BACH2-SNP that has recently been linked to a reduced expression of BACH2 protein and a more activated T cell phenotype of CD8$^+$ T cells in particular.[40,41] Our findings on CD4$^+$ T cells are complementary to this study and suggest that the examined SNPs do affect a wide range of different T cell subsets. In addition, we report on a potentially more severe phenotype of PSC in SNP carriers vs. non-carriers. However, given the lack of validated surrogate biomarkers in PSC[42] and the variable disease course, the presented clinical data are based on a rather limited number of genotyped participants and should be interpreted with caution. To generate robust clinical data, controlled multi-centre studies are needed. Overall, *rs56258221* represents only one of many polymorphisms contributing to the genetic risk in PSC. Moreover, as is true for most miRNAs, miR4464 has a variety of targets other than BACH2.[25] Therefore, we cannot exclude transfected miR4464 to target other molecules *in vitro*.

STAR★METHODS

Detailed methods are provided in the online version of this paper and include the following:

- KEY RESOURCES TABLE
- RESOURCE AVAILABILITY
  - Lead contact
  - Materials availability
  - Data and code availability
- EXPERIMENTAL MODEL AND STUDY PARTICIPANTS DETAILS
  - People with PSC and clinical data
- METHOD DETAILS
  - SNP genotyping
  - Isolation of PBMCs and CD4$^+$ T_N cells
  - Immunophenotyping
  - *In vitro* iT_{REG} polarization assay
  - *In vitro* T_H1 polarization assay
  - *In vitro* T_H17 polarization assay
  - *In vitro* proliferation assay
  - Real-time qPCR
  - FACS sorting and antibody staining for sequencing
  - Preparation of scRNA-Seq and CITE-Seq libraries
  - Sequencing
  - scRNA-Seq: data processing
  - scRNA-Seq: quality control and normalization
  - scRNA-Seq: ADT normalization
  - scRNA-Seq: Dimension reduction, clustering and differential expression analysis
  - Western Blot
  - Detection of miRNAs
  - Transfection of CD4$^+$ T_N with miR4464
  - RNA sequencing and analyses
  - BACH2 tester plasmid
  - Dual-Glo luciferase assay
- QUANTIFICATION AND STATISTICAL ANALYSIS
  - Statistical analysis

SUPPLEMENTAL INFORMATION

Supplemental information can be found online at https://doi.org/10.1016/j.xcrm.2024.101620.

ACKNOWLEDGMENTS

We want to thank all participants for taking part in our study and consenting to donate extra blood samples for research use. We thank Sabrina Kreß, Jennifer Wigger, Angela Schmidt, Marko Hilken, and Martina Fahl for excellent technical assistance.

This study was supported by the Deutsche Forschungsgemeinschaft (DFG; CRU306 290522633 and 426581255; LFF-FV78), the Helmut and Hannelore Greve Foundation, the YAEL Foundation (C. Schramm), and the Open Access Publication Fund of the University Medical Centre Hamburg-Eppendorf, Germany.

AUTHOR CONTRIBUTIONS

T.P. and J.B. conceptualized the study, performed the experiments, analyzed the data, and wrote the manuscript. J.K., I.E., H.G., L.K.K., L.A.L., N.I., A.-M.S., and A.L. performed the experiments and analyzed the data. C.C. performed analyses of sequencing data. S.S. and M.S. provided blood samples of the Hamburg PSC cohort. T.F., L.K.E., and T.H.K. provided blood samples and clinical data of the Oslo PSC cohort. A.F. provided the sequencing platform. C. Schlein, N.H., E.G., S.H., A.W.L., and N.G. advised the study and reviewed the manuscript. D.S. and C. Schramm conceptualized and supervised the study and wrote the manuscript.

DECLARATION OF INTERESTS

The authors declare no competing interests.

Received: August 12, 2023
Revised: April 16, 2024
Accepted: May 27, 2024
Published: June 19, 2024

REFERENCES

1. Karlsen, T.H., Folseraas, T., Thorburn, D., and Vesterhus, M. (2017). Primary sclerosing cholangitis - a comprehensive review. J. Hepatol. *67*, 1298–1323. https://doi.org/10.1016/j.jhep.2017.07.022.
2. Lazaridis, K.N., and LaRusso, N.F. (2016). Primary Sclerosing Cholangitis. N. Engl. J. Med. *375*, 1161–1170. https://doi.org/10.1056/NEJMra1506330.
3. Zhu, C., Boucheron, N., Müller, A.C., Májek, P., Claudel, T., Halilbasic, E., Baazim, H., Lercher, A., Viczenczova, C., Hainberger, D., et al. (2021). 24-Norursodeoxycholic acid reshapes immunometabolism in CD8(+) T cells

and alleviates hepatic inflammation. J. Hepatol. *75*, 1164–1176. https://doi.org/10.1016/j.jhep.2021.06.036.

4. Liaskou, E., Jeffery, L.E., Trivedi, P.J., Reynolds, G.M., Suresh, S., Bruns, T., Adams, D.H., Sansom, D.M., and Hirschfield, G.M. (2014). Loss of CD28 expression by liver-infiltrating T cells contributes to pathogenesis of primary sclerosing cholangitis. Gastroenterology *147*, 221–232.e7. https://doi.org/10.1053/j.gastro.2014.04.003.
5. Zimmer, C.L., von Seth, E., Buggert, M., Strauss, O., Hertwig, L., Nguyen, S., Wong, A.Y.W., Zotter, C., Berglin, L., Michaëlsson, J., et al. (2021). A biliary immune landscape map of primary sclerosing cholangitis reveals a dominant network of neutrophils and tissue-resident T cells. Sci. Transl. Med. *13*, eabb3107. https://doi.org/10.1126/scitranslmed.abb3107.
6. Kunzmann, L.K., Schoknecht, T., Poch, T., Henze, L., Stein, S., Kriz, M., Grewe, I., Preti, M., Hartl, J., Pannicke, N., et al. (2020). Monocytes as Potential Mediators of Pathogen-Induced T-Helper 17 Differentiation in Patients With Primary Sclerosing Cholangitis (PSC). Hepatology *72*, 1310–1326. https://doi.org/10.1002/hep.31140.
7. Sebode, M., Peiseler, M., Franke, B., Schwinge, D., Schoknecht, T., Wortmann, F., Quaas, A., Petersen, B.S., Ellinghaus, E., Baron, U., et al. (2014). Reduced FOXP3(+) regulatory T cells in patients with primary sclerosing cholangitis are associated with IL2RA gene polymorphisms. J. Hepatol. *60*, 1010–1016. https://doi.org/10.1016/j.jhep.2013.12.027.
8. Poch, T., Krause, J., Casar, C., Liwinski, T., Glau, L., Kaufmann, M., Ahrenstorf, A.E., Hess, L.U., Ziegler, A.E., Martrus, G., et al. (2021). Single-cell atlas of hepatic T cells reveals expansion of liver-resident naive-like CD4(+) T cells in primary sclerosing cholangitis. J. Hepatol. *75*, 414–423. https://doi.org/10.1016/j.jhep.2021.03.016.
9. Katt, J., Schwinge, D., Schoknecht, T., Quaas, A., Sobottka, I., Burandt, E., Becker, C., Neurath, M.F., Lohse, A.W., Herkel, J., and Schramm, C. (2013). Increased T helper type 17 response to pathogen stimulation in patients with primary sclerosing cholangitis. Hepatology *58*, 1084–1093. https://doi.org/10.1002/hep.26447.
10. Kummen, M., Thingholm, L.B., Ruhlemann, M.C., Holm, K., Hansen, S.H., Moitinho-Silva, L., Liwinski, T., Zenouzi, R., Storm-Larsen, C., Midttun, O., et al. (2021). Altered Gut Microbial Metabolism of Essential Nutrients in Primary Sclerosing Cholangitis. Gastroenterology *160*, 1784–1798.e1780. https://doi.org/10.1053/j.gastro.2020.12.058.
11. Liwinski, T., Zenouzi, R., John, C., Ehlken, H., Rühlemann, M.C., Bang, C., Groth, S., Lieb, W., Kantowski, M., Andersen, N., et al. (2020). Alterations of the bile microbiome in primary sclerosing cholangitis. Gut *69*, 665–672. https://doi.org/10.1136/gutjnl-2019-318416.
12. Karlsen, T.H., Franke, A., Melum, E., Kaser, A., Hov, J.R., Balschun, T., Lie, B.A., Bergquist, A., Schramm, C., Weismüller, T.J., et al. (2010). Genome-wide association analysis in primary sclerosing cholangitis. Gastroenterology *138*, 1102–1111. https://doi.org/10.1053/j.gastro.2009.11.046.
13. Melum, E., Franke, A., Schramm, C., Weismüller, T.J., Gotthardt, D.N., Offner, F.A., Juran, B.D., Laerdahl, J.K., Labi, V., Björnsson, E., et al. (2011). Genome-wide association analysis in primary sclerosing cholangitis identifies two non-HLA susceptibility loci. Nat. Genet. *43*, 17–19. https://doi.org/10.1038/ng.728.
14. Folseraas, T., Melum, E., Rausch, P., Juran, B.D., Ellinghaus, E., Shiryaev, A., Laerdahl, J.K., Ellinghaus, D., Schramm, C., Weismüller, T.J., et al. (2012). Extended analysis of a genome-wide association study in primary sclerosing cholangitis detects multiple novel risk loci. J. Hepatol. *57*, 366–375. https://doi.org/10.1016/j.jhep.2012.03.031.
15. Ellinghaus, D., Folseraas, T., Holm, K., Ellinghaus, E., Melum, E., Balschun, T., Laerdahl, J.K., Shiryaev, A., Gotthardt, D.N., Weismüller, T.J., et al. (2013). Genome-wide association analysis in primary sclerosing cholangitis and ulcerative colitis identifies risk loci at GPR35 and TCF4. Hepatology *58*, 1074–1083. https://doi.org/10.1002/hep.25977.
16. Liu, J.Z., Hov, J.R., Folseraas, T., Ellinghaus, E., Rushbrook, S.M., Doncheva, N.T., Andreassen, O.A., Weersma, R.K., Weismüller, T.J., Eksteen, B., et al. (2013). Dense genotyping of immune-related disease regions identifies nine new risk loci for primary sclerosing cholangitis. Nat. Genet. *45*, 670–675. https://doi.org/10.1038/ng.2616.
17. Ellinghaus, D., Jostins, L., Spain, S.L., Cortes, A., Bethune, J., Han, B., Park, Y.R., Raychaudhuri, S., Pouget, J.G., Hübenthal, M., et al. (2016). Analysis of five chronic inflammatory diseases identifies 27 new associations and highlights disease-specific patterns at shared loci. Nat. Genet. *48*, 510–518. https://doi.org/10.1038/ng.3528.
18. Liaskou, E., Jeffery, L., Chanouzas, D., Soskic, B., Seldin, M.F., Harper, L., Sansom, D., and Hirschfield, G.M. (2017). Genetic variation at the CD28 locus and its impact on expansion of pro-inflammatory CD28 negative T cells in healthy individuals. Sci. Rep. *7*, 7652. https://doi.org/10.1038/s41598-017-07967-2.
19. Muto, A., Hoshino, H., Madisen, L., Yanai, N., Obinata, M., Karasuyama, H., Hayashi, N., Nakauchi, H., Yamamoto, M., Groudine, M., and Igarashi, K. (1998). Identification of Bach2 as a B-cell-specific partner for small maf proteins that negatively regulate the immunoglobulin heavy chain gene 3' enhancer. EMBO J. *17*, 5734–5743. https://doi.org/10.1093/emboj/17.19.5734.
20. Labarta-Bajo, L., and Zúñiga, E.I. (2021). BAtCHing stem-like T cells during exhaustion. Nat. Immunol. *22*, 274–276. https://doi.org/10.1038/s41590-021-00891-8.
21. Roychoudhuri, R., Clever, D., Li, P., Wakabayashi, Y., Quinn, K.M., Klebanoff, C.A., Ji, Y., Sukumar, M., Eil, R.L., Yu, Z., et al. (2016). BACH2 regulates CD8(+) T cell differentiation by controlling access of AP-1 factors to enhancers. Nat. Immunol. *17*, 851–860. https://doi.org/10.1038/ni.3441.
22. Roychoudhuri, R., Hirahara, K., Mousavi, K., Clever, D., Klebanoff, C.A., Bonelli, M., Sciumè, G., Zare, H., Vahedi, G., Dema, B., et al. (2013). BACH2 represses effector programs to stabilize T(reg)-mediated immune homeostasis. Nature *498*, 506–510. https://doi.org/10.1038/nature12199.
23. Sidwell, T., Liao, Y., Garnham, A.L., Vasanthakumar, A., Gloury, R., Blume, J., Teh, P.P., Chisanga, D., Thelemann, C., de Labastida Rivera, F., et al. (2020). Attenuation of TCR-induced transcription by Bach2 controls regulatory T cell differentiation and homeostasis. Nat. Commun. *11*, 252. https://doi.org/10.1038/s41467-019-14112-2.
24. Yao, C., Lou, G., Sun, H.W., Zhu, Z., Sun, Y., Chen, Z., Chauss, D., Moseman, E.A., Cheng, J., D'Antonio, M.A., et al. (2021). BACH2 enforces the transcriptional and epigenetic programs of stem-like CD8(+) T cells. Nat. Immunol. *22*, 370–380. https://doi.org/10.1038/s41590-021-00868-7.
25. Chen, Y., and Wang, X. (2020). miRDB: an online database for prediction of functional microRNA targets. Nucleic Acids Res. *48*, D127–D131. https://doi.org/10.1093/nar/gkz757.
26. Tsukumo, S.i., Unno, M., Muto, A., Takeuchi, A., Kometani, K., Kurosaki, T., Igarashi, K., and Saito, T. (2013). Bach2 maintains T cells in a naive state by suppressing effector memory-related genes. Proc. Natl. Acad. Sci. USA *110*, 10735–10740. https://doi.org/10.1073/pnas.1306691110.
27. Edwards, C.L., de Oca, M.M., de Labastida Rivera, F., Kumar, R., Ng, S.S., Wang, Y., Amante, F.H., Kometani, K., Kurosaki, T., Sidwell, T., et al. (2018). The Role of BACH2 in T Cells in Experimental Malaria Caused by Plasmodium chabaudi chabaudi AS. Front. Immunol. *9*, 2578. https://doi.org/10.3389/fimmu.2018.02578.
28. Timperi, E., and Barnaba, V. (2021). CD39 Regulation and Functions in T Cells. Int. J. Mol. Sci. *22*, 8068. https://doi.org/10.3390/ijms22158068.
29. Crome, S.Q., Wang, A.Y., and Levings, M.K. (2010). Translational mini-review series on Th17 cells: function and regulation of human T helper 17 cells in health and disease. Clin. Exp. Immunol. *159*, 109–119. https://doi.org/10.1111/j.1365-2249.2009.04037.x.
30. Street, K., Risso, D., Fletcher, R.B., Das, D., Ngai, J., Yosef, N., Purdom, E., and Dudoit, S. (2018). Slingshot: cell lineage and pseudotime inference for single-cell transcriptomics. BMC Genom. *19*, 477. https://doi.org/10.1186/s12864-018-4772-0.
31. Roux de Bezieux, H., Van den Berge, K., Street, K., and Dudoit, S. (2024). Trajectory inference across multiple conditions with condiments. Nat. Commun. *15*, 833. https://doi.org/10.1038/s41467-024-44823-0.

CellPress
OPEN ACCESS

32. Bentsen, M., Goymann, P., Schultheis, H., Klee, K., Petrova, A., Wiegandt, R., Fust, A., Preussner, J., Kuenne, C., Braun, T., et al. (2020). ATAC-seq footprinting unravels kinetics of transcription factor binding during zygotic genome activation. Nat. Commun. *11*, 4267. https://doi.org/10.1038/s41467-020-18035-1.

33. Ji, S.G., Juran, B.D., Mucha, S., Folseraas, T., Jostins, L., Melum, E., Kumasaka, N., Atkinson, E.J., Schlicht, E.M., Liu, J.Z., et al. (2017). Genome-wide association study of primary sclerosing cholangitis identifies new risk loci and quantifies the genetic relationship with inflammatory bowel disease. Nat. Genet. *49*, 269–273. https://doi.org/10.1038/ng.3745.

34. Schoknecht, T., Schwinge, D., Stein, S., Weiler-Normann, C., Sebode, M., Mucha, S., Otto, B., Ellinghaus, E., Stahl, F., Franke, A., et al. (2017). CD4+ T cells from patients with primary sclerosing cholangitis exhibit reduced apoptosis and down-regulation of proapoptotic Bim in peripheral blood. J. Leukoc. Biol. *101*, 589–597. https://doi.org/10.1189/jlb.5A1015-469R.

35. Zhang, H., Dai, D., Hu, Q., Yang, F., Xue, Y., Li, F., Shen, N., Zhang, M., and Huang, C. (2021). Bach2 attenuates IL-2R signaling to control Treg homeostasis and Tfr development. Cell Rep. *35*, 109096. https://doi.org/10.1016/j.celrep.2021.109096.

36. Rutishauser, R.L., Martins, G.A., Kalachikov, S., Chandele, A., Parish, I.A., Meffre, E., Jacob, J., Calame, K., and Kaech, S.M. (2009). Transcriptional repressor Blimp-1 promotes CD8(+) T cell terminal differentiation and represses the acquisition of central memory T cell properties. Immunity *31*, 296–308. https://doi.org/10.1016/j.immuni.2009.05.014.

37. Jain, R., Chen, Y., Kanno, Y., Joyce-Shaikh, B., Vahedi, G., Hirahara, K., Blumenschein, W.M., Sukumar, S., Haines, C.J., Sadekova, S., et al. (2016). Interleukin-23-Induced Transcription Factor Blimp-1 Promotes Pathogenicity of T Helper 17 Cells. Immunity *44*, 131–142. https://doi.org/10.1016/j.immuni.2015.11.009.

38. Richer, M.J., Lang, M.L., and Butler, N.S. (2016). T Cell Fates Zipped Up: How the Bach2 Basic Leucine Zipper Transcriptional Repressor Directs T Cell Differentiation and Function. J. Immunol. *197*, 1009–1015. https://doi.org/10.4049/jimmunol.1600847.

39. Simerzin, A., Zorde-Khvalevsky, E., Rivkin, M., Adar, R., Zucman-Rossi, J., Couchy, G., Roskams, T., Govaere, O., Oren, M., Giladi, H., and Galun, E. (2016). The liver-specific microRNA-122*, the complementary strand of microRNA-122, acts as a tumor suppressor by modulating the p53/mouse double minute 2 homolog circuitry. Hepatology *64*, 1623–1636. https://doi.org/10.1002/hep.28679.

40. Mouri, K., Guo, M.H., de Boer, C.G., Lissner, M.M., Harten, I.A., Newby, G.A., DeBerg, H.A., Platt, W.F., Gentili, M., Liu, D.R., et al. (2022). Prioritization of autoimmune disease-associated genetic variants that perturb regulatory element activity in T cells. Nat. Genet. *54*, 603–612. https://doi.org/10.1038/s41588-022-01056-5.

41. Asimit, J.L., Rainbow, D.B., Fortune, M.D., Grinberg, N.F., Wicker, L.S., and Wallace, C. (2019). Stochastic search and joint fine-mapping increases accuracy and identifies previously unreported associations in immune-mediated diseases. Nat. Commun. *10*, 3216. https://doi.org/10.1038/s41467-019-11271-0.

42. Ponsioen, C.Y., Lindor, K.D., Mehta, R., and Dimick-Santos, L. (2018). Design and Endpoints for Clinical Trials in Primary Sclerosing Cholangitis. Hepatology *68*, 1174–1188. https://doi.org/10.1002/hep.29882.

43. Bolger, A.M., Lohse, M., and Usadel, B. (2014). Trimmomatic: a flexible trimmer for Illumina sequence data. Bioinformatics *30*, 2114–2120. https://doi.org/10.1093/bioinformatics/btu170.

44. Dobin, A., Davis, C.A., Schlesinger, F., Drenkow, J., Zaleski, C., Jha, S., Batut, P., Chaisson, M., and Gingeras, T.R. (2013). STAR: ultrafast universal RNA-seq aligner. Bioinformatics *29*, 15–21. https://doi.org/10.1093/bioinformatics/bts635.

45. Li, B., and Dewey, C.N. (2011). RSEM: accurate transcript quantification from RNA-Seq data with or without a reference genome. BMC Bioinf. *12*, 323. https://doi.org/10.1186/1471-2105-12-323.

46. Love, M.I., Huber, W., and Anders, S. (2014). Moderated estimation of fold change and dispersion for RNA-seq data with DESeq2. Genome Biol. *15*, 550. https://doi.org/10.1186/s13059-014-0550-8.

STAR★METHODS

KEY RESOURCES TABLE

REAGENT or RESOURCE	SOURCE	IDENTIFIER
Antibodies		
Brilliant Violet 605™ anti-human CD103 (clone: Ber-ACT8)	BioLegend	Cat: 350218; RRID: AB_2564283
PE/Dazzle™ anti-human CD11c (clone: 3.9)	BioLegend	Cat: 301641; RRID: AB_2564082
PE/Cyanine7 anti-human CD123 (clone: 6H6)	BioLegend	Cat: 306010; RRID: AB_493576
Brilliant Violet 650™ anti-human CD127 (clone: A019D5)	BioLegend	Cat: 351326; RRID: AB_2562095
Brilliant Violet 711™ anti-human CD14 (clone: M5E2)	BioLegend	Cat: 301838; RRID: AB_2562909
Brilliant Violet 605™ anti-human CD16 (clone: 9G8)	BioLegend	Cat: 302039; RRID: AB_2561354
APC/Cyanine7 anti-human CD16 (clone: 3G8)	BioLegend	Cat: 302018; RRID: AB_314218
PerCP/Cyanine5.5 anti-human CD160 (clone: BY55)	BioLegend	Cat: 341210; RRID: AB_2562874
PE anti-human CD161 (clone: HP-3G10)	BioLegend	Cat: 339904; RRID: AB_1501083
Brilliant Violet 605™ anti-human CD161 (clone: HP-3G10)	BioLegend	Cat: 339916; RRID: AB_2563607
Brilliant Violet 711™ anti-human CD183 (clone: G025H7)	BioLegend	Cat: 353732; RRID: AB_2563533
PE/Dazzle™ 594 anti-human CD19 (clone: HIB19)	BioLegend	Cat: 302252; RRID: AB_2563560
PE/Cyanine7 anti-human CD194 (clone: L291H4)	BioLegend	Cat: 359410; RRID: AB_2562431
PerCP/Cyanine5.5 anti-human CD196 (clone: G034E3)	BioLegend	Cat: 353406; RRID: AB_10918437
Alexa Fluor® 647 anti-human CD197 (clone: G043H7)	BioLegend	Cat: 353218; RRID: AB_10917385
Alexa Fluor® 647 anti-human CD199 (clone: L053E8)	BioLegend	Cat: 358911; RRID: AB_2562523
APC/Cyanine7 anti-human CD1c (clone: L161)	BioLegend	Cat: 331520; RRID: AB_10644008
Brilliant Violet 421™ anti-human CD20 (clone: 2H7)	BioLegend	Cat: 302330; RRID: AB_10965543
Alexa Fluor® 700 anti-human CD20 (clone: 2H7)	BioLegend	Cat: 302322; RRID: AB_493753
Alexa Fluor® 647 anti-human CD223 (clone: 11C3C65)	BioLegend	Cat: 369304; RRID: AB_2566480
PerCP/Cyanine5.5 anti-human CD24 (clone: ML5)	BioLegend	Cat: 311116; RRID: AB_10960741
Brilliant Violet 421™ anti-human CD25 (clone: BC96)	BioLegend	Cat: 302630; RRID: AB_11126749
Alexa Fluor® 647 anti-human CD268 (clone: 11C1)	BioLegend	Cat: 316914; RRID: AB_2203680
APC/Cyanine7 anti-human CD27 (clone: O323)	BioLegend	Cat: 302816; RRID: AB_571977
Brilliant Violet 421™ anti-human CD272 (clone: MIH26)	BioLegend	Cat: 344511; RRID: AB_2566507
Brilliant Violet 605™ anti-human CD279 (clone: EH12.2H7)	BioLegend	Cat: 329924; RRID: AB_2563212
PE/Cyanine7 anti-human CD28 (clone: CD28.2)	BioLegend	Cat: 302926; RRID: AB_10644005
PerCP/Cyanine5.5 anti-human CD3 (clone: OKT3)	BioLegend	Cat: 317336; RRID: AB_2561628
Brilliant Violet 650™ anti-human CD3 (clone: OKT3)	BioLegend	Cat: 317324; RRID: AB_2563352
Alexa Fluor® 488 anti-human CD38 (clone: HIT2)	BioLegend	Cat: 303512; RRID: AB_493088
PE/Cyanine7 anti-human CD39 (clone: A1)	BioLegend	Cat: 328212; RRID: AB_2099950
Alexa Fluor® 700 anti-human CD4 (clone: OKT4)	BioLegend	Cat: 317426; RRID: AB_571943
PE/Dazzle™ 594 anti-human CD4 (clone: RPA-T4)	BioLegend	Cat: 300548; RRID: AB_2563566
PE/Cyanine7 anti-human CD43 (clone: CD43-10G7)	BioLegend	Cat: 343208; RRID: AB_2563698
PE anti-human CD44 (clone: 338808)	BioLegend	Cat: 338808; RRID: AB_2076578
Brilliant Violet 785™ anti-human CD45 (clone: HI30)	BioLegend	Cat: 304048; RRID: AB_2563129
Brilliant Violet 711™ anti-human CD45RA (clone: HI100)	BioLegend	Cat: 304138; RRID: AB_2563815
PE/Cyanine7 anti-human CD49a (clone: TS2/7)	BioLegend	Cat: 328312; RRID: AB_2566272
FITC anti-human CD49b (clone: P1E6-C5)	BioLegend	Cat: 359306; RRID: AB_2562531
PE anti-human CD49d (clone: 9F10)	BioLegend	Cat: 304303; RRID: AB_314429
Brilliant Violet 421™ anti-human CD56 (clone: HCD56)	BioLegend	Cat: 318328; RRID: AB_11218798
PE/Dazzle™ 594 anti-human CD57 (clone: HNK-1)	BioLegend	Cat: 359619; RRID: AB_2564062
Brilliant Violet 510™ anti-human CD62L (clone: DREG-56)	BioLegend	Cat: 304843; RRID: AB_2617002

(*Continued on next page*)

Continued

REAGENT or RESOURCE	SOURCE	IDENTIFIER
PerCP/Cyanine5.5 anti-human CD69 (clone: FN50)	BioLegend	Cat: 310926; RRID: AB_2074956
APC/Cyanine7 anti-human CD69 (clone: FN50)	BioLegend	Cat: 310914; RRID: AB_314849
PE anti-human CD73 (clone: AD2)	BioLegend	Cat: 344004; RRID: AB_2298698
Brilliant Violet 510™ anti-human CD8a (clone: RPA-T8)	BioLegend	Cat: 301048; RRID: AB_2561942
PE/Dazzle™ 594 anti-human CD8a (clone: HIT8a)	BioLegend	Cat: 300930; RRID: AB_2629639
Alexa Fluor® 700 anti-human CD8a (clone: RPA-T8)	BioLegend	Cat: 301028; RRID: AB_493745
PE anti-human CD8b (clone: 2ST8.5H7)	BD Biosciences	Cat: 641057; RRID: AB_1645747
Alexa Fluor® 647 anti-human FOXP3 (clone: 259D)	BioLegend	Cat: 320214; RRID: AB_492984
FITC anti-human Granzyme B (clone: GB11)	BioLegend	Cat: 515403; RRID: AB_2114575
FITC anti-human HLA-DR (clone: L243)	BioLegend	Cat: 307604; RRID: AB_314682
APC/Cyanine7 anti-human IFN-γ (clone: 4S.B3)	BioLegend	Cat: 502530; RRID: AB_10663412
PE anti-human IL-10 (clone: JES3-9D7)	BioLegend	Cat: 501404; RRID: AB_315170
Brilliant Violet 711™ anti-human IL-17A (clone: BL168)	BioLegend	Cat: 512328; RRID: AB_2563888
PE/Cyanine7 anti-human IL-4 (clone: MP4-25D2)	BioLegend	Cat: 500824; RRID: AB_2126746
FITC anti-human/mouse integrin β7 (clone: FIB27)	BioLegend	Cat: 121010; RRID: AB_2129310
PE anti-human IgD (clone: IA6-2)	BioLegend	Cat: 348203; RRID: AB_10550096
PE-Cy™7 anti-auman TCRγ/δ (clone: 11F2)	BD Biosciences	Cat: 655410; RRID: AB_2870377
Brilliant Violet 650™ anti-human TNF-α (clone: MAb11)	BioLegend	Cat: 502938; RRID: AB_2562741
APC anti-human TCR Vα7.2 (clone: 3C10)	BioLegend	Cat: 351708; RRID: AB_10933246
FITC anti-human TCR delta (clone: TS-1)	Invitrogen	Cat: TCR2055; RRID: AB_223619
APC anti-human TCR Vd2 (clone: 123R3)	Miltenyi Biotec	Cat: 130-095-803; RRID: AB_10831200
FITC anti-human TCR-Vg9 (clone: IMMU 360)	BeckmanCoulter	Cat: IM1463; RRID: AB_130871
CITEseq: TotalSeq™-C0063 anti-human CD45RA (clone: HI100)	BioLegend	Cat: 304163; RRID: AB_2800764
CITEseq: TotalSeq™-C0087 anti-human CD45RO (clone: UCHL1)	BioLegend	Cat: 304259; RRID: AB_2800766
CITEseq: TotalSeq™-C0147 anti-human CD62L (clone: DREG-56)	BioLegend	Cat: 304851; RRID: AB_2800770
CITEseq: TotalSeq™-C0148 anti-human CD197 (clone: G043H7)	BioLegend	Cat: 353251; RRID: AB_2800943
CITEseq: TotalSeq™-C0124 anti-human CD31 (clone: WM59)	BioLegend	Cat: 303139; RRID: AB_2800757
CITEseq: TotalSeq™-C0154 anti-human CD27 (clone: O323)	BioLegend	Cat: 302853; RRID: AB_2800747
CITEseq: TotalSeq™-C0386 anti-human CD28 (clone: CD28.2)	BioLegend	Cat: 302963; RRID: AB_2800751
CITEseq: TotalSeq™-C0156 anti-human CD95 (clone: DX2)	BioLegend	Cat: 305651; RRID: AB_2800787
CITEseq: TotalSeq™-C0246 anti-human CD122 (clone: TU27)	BioLegend	Cat: 339021; RRID: AB_2814240
CITEseq: TotalSeq™-C0140 anti-human CD183 (clone: G025H7)	BioLegend	Cat: 353747; RRID: AB_2800949
CITEseq: TotalSeq™-C0185 anti-human CD11a (clone: TS2/4)	BioLegend	Cat: 350617; RRID: AB_2800935
CITEseq: TotalSeq™-C0576 anti-human CD49d (clone: 9F10)	BioLegend	Cat: 304345; RRID: AB_2814137
CITEseq: TotalSeq™-C0390 anti-human CD127 (clone: A019D5)	BioLegend	Cat: 351356; RRID: AB_2800937
CITEseq: TotalSeq™-C0085 anti-human CD25 (clone: BC96)	BioLegend	Cat: 302649; RRID: AB_2800745

(*Continued on next page*)

Continued

REAGENT or RESOURCE	SOURCE	IDENTIFIER
CITEseq: TotalSeq^{TM}-C0159 anti-human HLA-DR (clone: L243)	BioLegend	Cat: 307663; RRID: AB_2800795
CITEseq: TotalSeq^{TM}-C0901 anti-human GARP (clone: 7B11)	BioLegend	Cat: 352517; RRID: AB_2819994
CITEseq: TotalSeq^{TM}-C0176 anti-human CD39 (clone: A1)	BioLegend	Cat: 328237; RRID: AB_2800853
CITEseq: TotalSeq^{TM}-C0577 anti-human CD73 (clone: AD2)	BioLegend	Cat: 344031; RRID: AB_2800916
CITEseq: TotalSeq^{TM}-C0151 anti-human CD152 (clone: BNI3)	BioLegend	Cat: 369621; RRID: AB_2801015
CITEseq: TotalSeq^{TM}-C0360 anti-human CD357 (clone: 108-17)	BioLegend	Cat: 371227; RRID: AB_2810583
CITEseq: TotalSeq^{TM}-C0171 anti-human CD278 (clone: C398.4A)	BioLegend	Cat: 313553; RRID: AB_2800823
CITEseq: TotalSeq^{TM}-C0071 anti-human CD194 (clone: L291H4)	BioLegend	Cat: 359425; RRID: AB_2800988
CITEseq: TotalSeq^{TM}-C0143 anti-human CD196 (clone: G034E3)	BioLegend	Cat: 353440; RRID: AB_2810563
CITEseq: TotalSeq^{TM}-C0141 anti-human CD195 (clone: J418F1)	BioLegend	Cat: 359137; RRID: AB_2810570
CITEseq: TotalSeq^{TM}-C0366 anti-human CD184 (clone: 12G5)	BioLegend	Cat: 306533; RRID: AB_2800791
CITEseq: TotalSeq^{TM}-C0144 anti-human CD185 (clone: J252D4)	BioLegend	Cat: 356939; RRID: AB_2800968
CITEseq: TotalSeq^{TM}-C0125 anti-human CD44 (clone: BJ18)	BioLegend	Cat: 338827; RRID: AB_2800900
CITEseq: TotalSeq^{TM}-C0146 anti-human CD69 (clone: FN50)	BioLegend	Cat: 310951; RRID: AB_2800810
CITEseq: TotalSeq^{TM}-C0149 anti-human CD161 (clone: HP-3G10)	BioLegend	Cat: 339947; RRID: AB_2810532
CITEseq: TotalSeq^{TM}-C0581 anti-human TCR Vα7.2 (clone: 3C10)	BioLegend	Cat: 351735; RRID: AB_2810556
CITEseq: TotalSeq^{TM}-C0088 anti-human CD279 (clone: EH12.2H7)	BioLegend	Cat: 329963; RRID: AB_2800862
CITEseq: TotalSeq^{TM}-C0378 anti-mouse CD223 (clone: C9B7W)	BioLegend	Cat: 125237; RRID: AB_2832450
CITEseq: TotalSeq^{TM}-C0250 anti-mouse/human KLRG1 (clone: 2F1/KLRG1)	BioLegend	Cat: 138433; RRID: AB_2800649
CITEseq: TotalSeq^{TM}-C0168 anti-human CD57 (clone: QA17A04)	BioLegend	Cat: 393321; RRID: AB_2801030
CITEseq: TotalSeq^{TM}-C0169 anti-human CD366 (clone: F38-2E2)	BioLegend	Cat: 345049; RRID: AB_2800925
CITEseq: TotalSeq^{TM}-C0089 anti-human TIGIT (clone: A15153G)	BioLegend	Cat: 372729; RRID: AB_2801021
CITEseq: TotalSeq^{TM}-C0139 anti-human TCR γ/δ (clone: B1)	BioLegend	Cat: 331231; RRID: AB_2814199
CITEseq: TotalSeq^{TM}-C0584 anti-human TCR Vα24-Jα18 (clone: 6B11)	BioLegend	Cat: 342925; RRID: AB_2810539
Western Blot Antibody: BACH2	Cell Signaling Technology	Cat: 80775; RRID: AB_2799961
Western Blot Antibody: beta-Actin (C4)	Cell Signaling Technology	Cat: SC-47778; RRID: AB_626632
anti-human CD3 antibody; clone: OKT3	BioLegend	Cat: 317302; RRID: AB_571927
anti-huamn CD28 antibody; clone: CD28.1	BioLegend	Cat: 302934; RRID: AB_2616667

(*Continued on next page*)

Continued		
REAGENT or RESOURCE	SOURCE	IDENTIFIER
anti-human IL-4 antibody	Miltenyi Biotech	Cat: 130-095-753; RRID: AB_10831210
anti-human IFNγ antibody	Miltenyi Biotech	Cat: 130-095-743; RRID: AB_10830868
anti-human IL-12 antibody	PeproTech	Cat: 500-P154G; RRID: AB_2929517
**Biological samples**		
Human peripheral blood (german cohort)	University Medical Center Hamburg-Eppendorf (I. Dept. of Medicine) (Hamburg, Germany)	N/A
Liver tissue for bulk RNA seq	University Medical Center Hamburg-Eppendorf (I. Dept. of Medicine) (Hamburg, Germany)	N/A
Human peripheral blood (norwegian cohort)	Norwegian PSC Center (Oslo, Norway)	N/A
**Chemicals, peptides, and recombinant proteins**		
IL-2 (cell culture)	R&D Systems	Cat: 202-IL-010
ATRA (cell culture)	Sigma-Aldrich	Cat: R2625
TGFβ (cell culture)	Miltenyi Biotech	Cat: 130-095-066
IL-6 (research grade)	Miltenyi Biotech	Cat: 130-093-929
IL-1β (research grade)	Miltenyi Biotech	Cat: 130-095-374
IL-23 (research grade)	Miltenyi Biotech	Cat: 130-095-758
IL-7	Miltenyi Biotech	Cat: 130-095-362
RPMI 1640 medium	Gibco	Cat: 11875093
Opti-MEM medium	Gibco	Cat: 31985070
Fetal calve serum (FCS/FBS)	PAN Biotech	Cat: P30-3033
1% Penicillin-Streptomycin	Sigma Aldrich	Cat: P4333
Fixable Viability Dye eFluor 506	ThermoFisher	Cat: 65-0866-14
Bovine serum albumine (BSA)	Carl Roth	Cat: 0163.4
Phosphate buffered saline (PBS)	Gibco	Cat: 10010023
Phorbol 12-myristate 13-acetate (PMA)	Sigma Aldrich	Cat: P1585
Ionomycin	Sigma Aldrich	Cat: I24222
Brefeldin A	BD Bioscience	Cat: 55029
**Critical commercial assays**		
TaqMan SNP Genotyping assay (rs56258221)	ThermoFisher	Cat: C__88670967_10
TaqMan SNP Genotyping assay (rs80060485)	ThermoFisher	Cat: C_103476844_10
TaqMan SNP Genotyping assay (rs4147359)	ThermoFisher	Cat: C___1841422_10
TaqMan SNP Genotyping assay (rs7426056)	ThermoFisher	Cat: C__29052378_10
CellTrace Violet Cell Proliferation Kit	ThermoFisher	Cat: C34557
DNA Blood Midi Kit	Qiagen	Cat: 51104
Ficoll-Paque PLUS density gradient media	Cytiva	Cat: 17144003
MACS: Naive CD4+ T cell Isolation Kit II, human	MIltenyi Biotec	Cat: 130-094-131
Foxp3/Transcription Factor Staining Buffer Set	eBioscience/Invitrogen	Cat: 00-5523-00
NucleoSpin RNA Kit	Macherey-Nagel	Cat: 740955.250
High-Capacity cDNA Reverse Transcription Kit	Applied Biosystems	Cat: 4368813
KAPA PROBE FAST 1PCR Kit	KAPA Biosystems	Cat: KK4715
HPRT housekeeper	ThermoFisher	Cat: Hs02800695_m1
Chromium Next GEM Single Cell 5′ Reagent Kit v2	10x Genomics	Cat: 1000265
Chromium Next GEM Chip K Single Cell Kit	10x Genomics	Cat: 1000287

(Continued on next page)

Continued		
REAGENT or RESOURCE	SOURCE	IDENTIFIER
Dynabeads MyOne SILAN	ThermoFisher	Cat: 37002D
TaqMan MicroRNA Cells-to-C_T Kit	ThermoFisher	Cat: 4391848
TaqMan MicroRNA Assay for miR4464	ThermoFisher	Cat: 463103_mat
TaqMan MicroRNA Control Assay (RNU48)	ThermoFisher	Cat: 001006
Amaxa Cell Line Nucleofactor Kit V	Lonza Bioscience	Cat: VCA-1003
Transit-X2-Dynamic delivery system	Mirusbio	Cat: MIR 6004
Dual-Luciferase Reporter Assay System	Promega	Cat: E1910
Deposited data		
CITE-sequencing data	This paper	ArrayExpress: E-MTAB-14013
Bulk RNAseq data	This paper	ArrayExpress: E-MTAB-14103
Experimental models: Cell lines		
HEK293T cells	ATCC	Cat: CRL-3216; RRID: CVCL_0063
Oligonucleotides		
miR4464 (for transfection experiment)	ThermoFisher	ID: MC22259
non-binding (mock) miR	ThermoFisher	ID: 4464058
Recombinant DNA		
BACH2-miR4464 tester plasmids	This paper	Table S4
pmiR-GLO dual luciferase reporter vector	Promega	Cat: E1330
Software and algorithms		
FlowJo v10	https://www.flowjo.com	RRID: SCR_008520
Graphpad Prism 9.3	https://www.graphpad.com	RRID: SCR_002798
R 3.6.2	The R Foundation https://www.r-project.org/	RRID: SCR_001905
Seurat 3.1	https://satijalab.org/seurat/	RRID: SCR_07322
Cellranger 3.0.2	10x Genomics https://support.10xgenomics.com/single-cell-gene-expression/software/pipelines/latest/what-is-cell-ranger	RRID: SCR_017344
tidyverse 2.0.0	https://www.tidyverse.org/	RRID: SCR_019186
Other		
ViiA 7 Real-Time PCR System	Applied Biosystems	Cat: 4453536
LSRFortessa	BD Biosciences	RRID: SCR_018655
BD FACS Aria III	BD Biosciences	RRID: SCR_016695
Cytek Aurora	Cytek	Part Number: N7-00003
2100 Bioanalyzer Instrument	Agilent Technologies	RRID: SCR_018043
QuBit 3.0 Fluorometer	ThermoFisher	RRID: SCR_020311
NovaSeq6000	Illumina	RRID: SCR_016387
Nucleofector 2b Device	Lonza Bioscience	RRID: SCR_022262
BioTek Synergy H1 microplate luminometer	Agilent	Cat: H1MG

RESOURCE AVAILABILITY

Lead contact

Further information and requests for resources and reagents should be directed to and will be fulfilled by the lead contact, Christoph Schramm (c.schramm@uke.de).

Materials availability

All unique/stable reagents generated in this study are available from the lead contact with a completed Materials Transfer Agreement.

Data and code availability

- (Section 1: Data) Processed single-cell-sequencing data have been deposited at the ArrayExpress database (European Bioinformatics Institute) and are publicly available as of the date of publication. Accession numbers are listed in the key resources table. Raw sequencing data reported in this study cannot be deposited in a public repository due to data privacy concerns. Original western blot images and de-identified bulk RNAseq data will be shared by the lead contact upon request.
- (Section 2: Code) This paper does not report original code.
- (Section 3: Statement) Any additional information required to reanalyze the data reported in this paper is available from the lead contact upon request.

EXPERIMENTAL MODEL AND STUDY PARTICIPANTS DETAILS

People with PSC and clinical data

We conducted a cross-sectional study in adult people (>18 years of age) with PSC either at the University Medical Centre Hamburg-Eppendorf (Hamburg, Germany) (n = 210) or Oslo University Hospital Rikshospitalet (Oslo, Norway) (n = 138). Fresh blood samples from people with PSC were collected via the YAEL outpatient service of the I. Department of Medicine. Gender and age matched healthy blood donors (HD) were used as controls. Liver tissue for bulk RNAseq was obtained from people with PSC who had undergone biopsy at the I. Department of Medicine (University Medical Center Hamburg-Eppendorf). The biopsies were taken during mini-laparoscopy by using TruCut needles. Immediatly after material extraction the tissue was preserved in liquid nitrogen.

All study participants provided written informed consent according to the ethical guidelines of the Institutional Review Board of the medical faculty of the University of Hamburg (PV4081).

METHOD DETAILS

SNP genotyping

Genomic DNA was isolated from peripheral venous blood or serum by using the *Qiagen DNA Blood Midi Kit* (Qiagen, NL) according to the manufacturer's instructions. TaqMan 5′-nuclease assays were performed as 10 μL-reactions on 96-well plates using TaqMan Genotyping Mastermix and predesigned TaqMan probes. Each run consisted of a 10 min hold cycle at 95°C, followed by 40 cycles of 95°C for 15 s and 60°C for 1 min. Cycling and detection of reporter signals (VIC/FAM) were performed using a ViiA 7 Real-Time PCR System (Applied Biosystems, USA).

Isolation of PBMCs and $CD4^+$ T_N cells

Blood samples were collected in EDTA tubes and stored overnight at 4°C. Peripheral Blood Mononuclear Cells (PBMCs) were isolated by density gradient centrifugation (Ficoll-Paque PLUS, Cytiva, UK). Subsequently, $CD4^+$ T_N cells were isolated from PBMCs using the *Naive CD4⁺ T cell Isolation Kit II*, *human* (Miltenyi Biotec, GER) and purity was determined by flow cytometry ($CD4^+$ $CD197^+$ $CD45RA^+$ cells). Only samples with a purity of ≥90% T_N of total $CD4^+$ were used for further experiments.

Immunophenotyping

Heparinized whole blood of people with PSC, PBC, IBD, NAFLD or healthy donors was stained with 8 different antibody cocktails for 30 min at room temperature. Afterward, RBC Lysis/Fixation solution (Biolegend, USA) was added and after 10 min cells were subsequently washed two times with PBS. Stained cells were suspended in PBS containing 2% FCS and 0.01% NaN_3. Samples were measured using a BD LSRFortessa (BD Biosciences, USA) or Cytek Aurora (Cytek) Following analyses was performed using FlowJo v10 (BD Biosciences, USA).

In vitro iT_{REG} polarization assay

$CD4^+$ T_N cells were cultured in the presence of coated anti-CD3 (2 μg/mL, OKT3, Biolegend, USA), anti-CD28 (1 μg/mL, CD28.2, Biolegend, USA), IL-2 (100 U/ml, R&D Systems, USA), all-trans retinoic acid (ATRA, 10 nM, Sigma Aldrich, GER) and TGF-β1 (7 ng/mL, Miltenyi Biotec, GER) in RPMI1640 medium (Gibco) containing 10% fetal calf serum (FCS) (PAN Biotech, GER) and 1% Penicillin-Streptomycin (Sigma Aldrich, GER). On day 3, medium and cytokines were substituted. Cells were analyzed on day 6 by flow cytometry. Staining was performed by using the *eBioscience Foxp3/Transcription Factor Staining Buffer Set* (eBioscience, USA). T_{REG} were defined as $CD4^+$ $CD25^+$ $CD127^{lo}$ $CD152^+$ $Foxp3^+$ cells.

In vitro T_H1 polarization assay

$CD4^+$ T_N cells were cultured in the presence of coated anti-CD3 (2 μg/mL, OKT3, Biolegend, USA), anti-CD28 (1 μg/mL, CD28.2, Biolegend, USA), anti-IL-4 (2.5 μg/mL, Miltenyi Biotec, GER), IL-2 (100 U/ml, R&D Systems, USA) and IL-12 (375 U/ml, Miltenyi Biotec, GER) in RPMI1640 medium (Gibco, USA) containing 10% fetal calf serum (FCS) (PAN Biotech, GER) and 1% Penicillin-Streptomycin (Sigma Aldrich, GER). On day 3, medium and cytokines were substituted. Cells were analyzed on day 6 by flow cytometry. Before staining, cells were restimulated with Phorbol 12-myristate 13-acetate (50 ng/ml) (Sigma Aldrich), Ionomycin (1μg/ml) (Sigma Aldrich)

and Brefelding A (1x) (BD Bioscience) for 4 h at 37°. Intracellular staining was performed by using the *eBioscience Foxp3/ Transcription Factor Staining Buffer Set* (eBioscience, USA). T_H1 cells were defined as $CD4^+$ $TNF\alpha^+$ $IFN\gamma^+$ cells.

In vitro T_H17 polarization assay

$CD4^+$ T_N cells were cultured in the presence of coated anti-CD3 (2 μg/mL, OKT3, Biolegend, USA), anti-CD28 (1 μg/mL, CD28.2, Biolegend, USA), anti-IL-4 (2.5 μg/mL), anti-IFNγ (1 μg/mL, both Miltenyi Biotec, GER), anti-IL-12 (1 μg/mL, PeproTech, GER), IL-6, IL-1b and IL-23 (all 30 ng/mL and all Miltenyi Biotec, GER) in RPMI1640 medium (Gibco, USA) containing 10% fetal calf serum (FCS) (PAN Biotech, GER) and 1% Penicillin-Streptomycin (Sigma Aldrich, GER). On days 5 and 8, medium and cytokines were substituted. Cells were analyzed on day 12 by flow cytometry. T_H17 cells were defined as $CD4^+$ $IL\text{-}17A^+$ cells.

In vitro proliferation assay

Isolated $CD4^+$ T_N cells were stained with the *CellTrace Violet Cell Proliferation Kit* (ThermoFisher Scientific, USA) according to the manufacturer's protocol and cultured in the presence of coated anti-CD3 (2 μg/mL, OKT3, Biolegend, USA), anti-CD28 (1 μg/mL, CD28.2, Biolegend, USA) and IL-7 (500 U/ml, Miltenyi Biotec, GER) in RPMI1640 medium (Gibco, USA) containing 10% fetal calf serum (FCS) (PAN Biotech, GER) and 1% Penicillin-Streptomycin (Sigma Aldrich, GER). Cells were analyzed on day 6 by flow cytometry.

Real-time qPCR

RNA was extracted using the *NucleoSpin RNA Kit* (Macherey-Nagel, GER) according to the manufacturer's protocol. 0.7μg of extracted RNA was transcribed into cDNA using *High-Capacity cDNA Reverse Transcription Kit* (Applied Biosystems, USA). qRT-PCR was performed using *KAPA PROBE FAST 1PCR Kit* and *KAPA PROBE FAST ROX low* (KAPA Biosystems, UK). Expression of target genes was normalized to the expression of *hypoxanthin-guanine phosphoribosyltransferase* (HPRT) (02800695_m1, ThermoFisher Scientific, USA) as a housekeeper. Expression data was examined using the $2^{-\Delta Ct}$ method.

FACS sorting and antibody staining for sequencing

Peripheral blood $CD3^+$ T cells were isolated and FACS-sorted from previously cryopreserved peripheral blood mononuclear cells (PBMCs). Previously frozen cells were thawed and diluted with 50 mL of RPMI containing 10% FCS (PAN Biotech, GER) and 1% P/S (Sigma Aldrich, GER). After centrifugation, cells were washed with PBS and then stained with FITC-conjugated anti-CD3 antibody at a 1:200 dilution in combination with live/dead staining using Fixable Viability Dye eFluor 506 (Amcyan) (ThermoFisher Scientific, USA) at a dilution of 1:2000. At the same time, cells were stained with oligo-conjugated antibodies for CITE-Seq using TotalSeq A antibodies, (BioLegend, USA). Staining of cells was performed for 30 min on ice and after washing the cells, subsequent FACS-sorting and utilization of the *Chromium Single-Cell platform* (10x Genomics, USA) was performed.

Preparation of scRNA-Seq and CITE-Seq libraries

The scRNA-Seq library was prepared using the *Chromium Next GEM Single Cell 5′ Reagent Kit v2*, according to the manufacturer's instructions (10x Genomics, USA). FACS-sorted cells were washed once with PBS containing 0.04% bovine serum albumin (BSA) and resuspended in PBS. 20,000 cells were used for GEM generation through the 10x Chromium Controller using the *Chromium Next GEM Chip K Single Cell Kit* (10x Genomics, USA). Briefly, droplet preparation was followed by reverse transcription and cell barcoding, the emulsions were resolved and cDNA was purified using Dynabeads MyOne SILANE (ThermoFisher Scientific, USA) followed by a PCR amplification with additional use of a primer for amplification of antibody derived tags (ADTs). Amplified cDNA was then used for construction of 5′ gene expression library, V(D)J-library and ADT library using the dual index strategy following the manufacturer's instructions. Quality control and quantification of the generated libraries was conducted using a 2100 Bioanalyzer Instrument (Agilent Technologies, USA) and a QuBit 3.0 Fluorometer (ThermoFisher Scientific, USA), respectively.

Sequencing

The libraries were sequenced on an Illumina NovaSeq6000 to a minimum sequencing depth of 20,000 reads per cell for gene expression library, as well as a minimum sequencing depth of 5,000 reads for TCR- and ADT-libraries using read lengths of 100 bp read 1 (26 cycles), 8bp i5 index (10 cycles), 8 bp i7 index (10 cycles), 100 bp read 2 (90 cycles).

scRNA-Seq: data processing

For the scRNA-Seq data, after demultiplexing, reads were aligned against the GRCh38 human reference genome (release 93) and summarized using the Cellranger pipeline (version 3.0.2, 10x Genomics, USA). Further analysis steps were performed in R (version 3.6.2; The R Foundation for Statistical Computing, AUT).

scRNA-Seq: quality control and normalization

For each sample, genes not observed in at least 1% of all cells were dropped. Low quality or damaged cells were excluded using a combination of multiple sample dependent quality measures: minimum UMI count (mean 1234, range 800–2000), minimum and maximum number of expressed genes (mean 756, range 450–1000, and mean 2625, range 2500–3000 respectively), and

mitochondrial transcript percentage (mean 6%, range 5–7.5%). Additionally, we filtered out doublet cell candidates using *Scrublet* (*41*), adjusting its estimated doublet threshold as needed.

For normalization we used Seurat's SCTransform (*40*) function. SCTransform combines the usual scRNA workflow of data normalization, identification of highly variable genes (HVG) and data scaling. Briefly, for each gene a regularized negative binomial regression is performed and the resulting pearson residuals (regression residuals divided by expected standard deviation) represent a variance-stabilizing transformation of the expression data that can be used as normalization for downstream analysis. HVGs are selected based on the highest variance in the pearson residuals.

We opted not to correct for possible cell cycle influence during this process since visual inspection of the first two principal components on cell cycle genes (cc.genes included in Seurat) did not show an obvious bias toward these genes. These steps resulted in overall 8 (SNP-carrier and non-carrier, $n = 4$ each) samples and 56209 cells (SNP-carrier: 31014; non-carrier: 25195).

scRNA-Seq: ADT normalization

For each sample where ADT expression data was available, we applied feature-wise centered log ratio transformation implemented in Seurat's NormalizeData function.

scRNA-Seq: Dimension reduction, clustering and differential expression analysis

The batched corrected counts for all integration anchors were used as input for PCA. For UMAP embedding and graph-based shared nearest neighbor clustering we used up to 20 principle components. We iteratively increased the clustering resolution parameter until no additional clusters with biological meaningful cluster markers were detected. Cluster marker detection was performed by differential gene expression analysis for each cluster against all remaining cells using logistic regression on the sample-wise normalized RNA matrices. We included the donor variable as covariate in the regression model and tested for significant differential expression against a null model with likelihood ratio test.

Cluster labels were assigned using a combination of detected marker genes, ADT expression and UMAP overlay of the cell populations identified by manual gating of the ADT expression.

We identified 16 clusters, the biggest clusters expectedly comprising naive $CD4^+$ T cells (C0: $CD4^+$ T_N, C1: $CD4^+$ T_N mature), identified by high expression of *CCR7*, *SELL*, *TCF7* and *FOXP1*. Central memory $CD4^+$ T cells (C2: $CD4^+$ T_{CM}) were identified by *ICOS*, *MAL*, *SELL*, *IL7R* mRNA and CCR7 protein expression. A variety of different T Helper (T_H) cell subtypes (C10: $CD4^+$ T_H1: *CXCR3*, *PRDM1*, *CD69*, *STAT1*; C7: $CD4^+$ T_H2: *GATA3*, *CD69*, *CCR4*, *TNFRSF4*; C11: $CD4^+$ T_H17: *KLRB1*, *CCR6*, *RORA*, *PRDM1*) as well as two clusters of regulatory T cells (C12: $CD4^+$ T_{REG} naive: *FOXP3*, *FOXP1*, *IL2RA*, *SELL*; C14: $CD4^+$ T_{REG} effector: *FOXP3*, *LGALS3*, *CTLA4*, *HLA-DRB1*) (Figures S1H–S1I). Naive $CD8^+$ T cells (C3: $CD8^+$ T_N) were identified by high expression of *CCR7*, *SELL*, *PECAM1*, *TCF7*. Effector memory $CD8^+$ T cells (C4: $CD8^+$ T_{EM}) were identified by the expression of *KLRD1*, *NKG7*, *PRF1* and terminally differentiated $CD8^+$ T cells (C5: $CD8^+$ T_C term. diff.) by the additional expression of *CX3CR1* and *ZEB2*. Type-1 cytotoxic $CD8^+$ T cells (C6: $CD8^+$ T_C1) were identified by the expression of *CXCR3*, *KLRG1*, *EOMES* and NK-like $CD8^+$ T cells (C8: $CD8^+$ T_C NK-like) by *GNLY*, *GZMB*, *FCGR3A*. In addition, natural killer T cells (C9: NKT) were identified by similar markers as C8, but expressing CD4 instead of CD8. Other unconventional T cell subsets, as gamma delta T cells (C13: $\gamma\delta$ T cells) were identified by the high expression of *TRDV2*, *TRGV9*, *NKG7* and *PRF1*, whereas mucosa-associated invariant T cells (MAIT) were identified by the high expression of *KLRB1*, *CCR6*, *RORC* and *ZBTB16*.

Western Blot

To determine protein levels of BACH2 in FACS-sorted T_N cells, we washed cells and resuspended the pellet in glycerol-based lysis buffer including protease inhibitors. After sonification, we determined the protein content and denaturated samples by addition of the respective volume of 5x Laemmli buffer and subsequent heating to 95°C for 5 min. For gel electrophoresis, we used 12% SDS-gels and used 10 μg total protein. For protein transfer on nitrocellulose membranes, we used the wet blotting system. After blocking steps with 5% non-fat dried milk in TBST, we used antibodies against BACH2 (Cell Signaling Technology, USA) and beta-Actin (Cell Signaling Technology, USA).

Detection of miRNAs

To detect miRNAs, we used the Cells-to-C_T Kit (ThermoFisher Scientific, USA) on 10^5 or less FACS-sorted T_N cells per reaction, according to the manufacturer's recommendations and using Single-Primer reactions. To detect the miRNAs of interest, we used TaqMan-based probes against miR4464 (ThermoFisher Scientific, USA) and normalized its expression to ubiquitously expressed RNU48 (ThermoFisher Scientific, USA).

Transfection of $CD4^+$ T_N with miR4464

For transfection of $CD4^+$ T_N from HD and people with PSC, we used freshly isolated $CD4^+$ T_N from peripheral blood, as described above. We seeded $3x10^5$ cells per well in 96-well plates, previously coated with 2 μg/mL anti-CD3 antibodies (clone OKT3, Biolegend, USA), and incubated the cells under serum-free conditions for 4 days in Opti-MEM (Gibco, USA) containing 1 μg/mL anti-CD28 antibodies (clone CD28.1, Biolegend, USA). For transfection, we harvested the cells, counted them and used $2x10^6$ or less cells per transfection, using the Amaxa Cell Line Nucleofector Kit V (Lonza Bioscience, SUI) in combination with the Nucleofector 2b

Device (Lonza Bioscience, SUI), according to the manufacturer's protocol. For each transfection we used miR4464 or mock control at 1 nM. After transfection, we seeded the cells again into 96-well plates coated with 2 μg/mL anti-CD3 antibodies (clone OKT3, Biolegend, USA) in RPMI1640 medium (Gibco,USA) containing 10% fetal calf serum (PAN Biotech, GER), 1% Penicillin-Streptomycin (Sigma Aldrich, GER) and 100 U/ml IL-2 (R&D Systems, USA). After 24 h, we changed to medium to iT_{REG} polarizing conditions and performed further culture and analyses as described above.

RNA sequencing and analyses

RNA quality was assessed using Bioanalyzer, and samples with and RNA integrity number >7 were included for RNA sequencing. Up to 1ug RNA was used to synthesize mRNA libraries using TruSeq stranded mRNA library Preparation Kit (Illumina) on a Hiseq 4000 system (Illumina).

We used FastQC (version 0.11.5, Babraham Institute, United Kingdom) for a general quality check of the raw fastq files. TruSeq2-PE adapter and low quality read trimming was performed with Trimmomatic[43] (version 0.36) using the options ILLUMINACLIP: TruSeq2-PE.fa:2:30:10:2:falseSLIDINGWINDOW:4:15. Subsequently, the reads were aligned against the ensemble 87 reference genome and ensemble 87 reference annotation with STAR (version 2.7.3a).[44] On average read depth for the 47 samples was 40.310.540 (range: 8.318.152–61.856.384) and on average 92.76% (range: 79.27%–95.88%) of the reads were uniquely mapped to the reference genome. TPM were estimated using RSEM[45] (v 1.3.0).

Further analysis was performed with R (version 1.2.1335, The R Foundation for Statistical Computing, Austria). To filter the data, a threshold of ≥10 counts in all samples was set. We used DESeq2 for normalization and differential gene expression analysis.[46] Data was inspected for possible confounding effects using PCA based on regularized logarithm transformed counts.

BACH2 tester plasmid

In order to generate the wild type plasmid, a fragment of the BACH2-3′UTR containing the predicted miR4464 binding site (Fr1) was created by PCR using primers Pr1 and Pr2 (Table S7). The predicted miR4464 binding site was obtained from the miRDB database (*25*). The fragment carrying the mutated miR4464 site in the BACH2-3′UTR sequence (Fr2) was created by two PCR reactions: the first performed with Pr1 and Pr4 and the second performed with the product of the first PCR as forward primer and Pr2 (Table S7). The PCR products were then cloned into the *pmirGLO dual luciferase reporter vector* (Promega, USA) downstream the firefly luciferase gene.

Dual-Glo luciferase assay

HEK293T cells were transfected with 40ng of wildtype or mutated plasmid together with 61,6nM of miR4464 (ThermoFisher Scientific, USA) or control miR (ThermoFisher Scientific, USA) using Mirus transfection reagent (*Transit-X2-Dynamic delivery system*). After 48 h, cells were harvested for measuring luciferase activity as well as protein content. Luciferase activity was measured in a microplate luminometer (Synergy H1 BioTek) using the *Dual-Luciferase Reporter Assay System* (Promega, USA) following the manufacturer's protocol. The indicated values represent firefly luciferase activities normalized to the Renilla luciferase activities.

QUANTIFICATION AND STATISTICAL ANALYSIS

Statistical analysis

Statistical analysis was performed using GraphPad Prism software Version 9.3 (GraphPad Software, USA). Differences between two groups were analyzed by unpaired t-Test with Welch's correction (normal distribution) or by Mann-Whitney test (no normal distribution). Differences between 3 groups or more were evaluated using the ordinary one-way ANOVA (normal distribution) or Kruskal-Wallis test (no normal distribution). *p*-Values below 0.05 were considered statistically significant. Data is presented as median ± interquartile range, unless otherwise stated. Categorial data (e.g., LTX-frequency) between two groups were analyzed using contingency tables with Fisher's exact test.

Cell Reports Medicine, Volume *5*

Supplemental information

Intergenic risk variant *rs56258221* skews the fate of naive $CD4^+$ T cells via miR4464-BACH2 interplay in primary sclerosing cholangitis

Tobias Poch, Jonas Bahn, Christian Casar, Jenny Krause, Ioannis Evangelakos, Hilla Gilladi, Lilly K. Kunzmann, Alena Laschtowitz, Nicola Iuso, Anne-Marie Schäfer, Laura A. Liebig, Silja Steinmann, Marcial Sebode, Trine Folseraas, Lise K. Engesæter, Tom H. Karlsen, Andre Franke, Norbert Hubner, Christian Schlein, Eithan Galun, Samuel Huber, Ansgar W. Lohse, Nicola Gagliani, Dorothee Schwinge, and Christoph Schramm

SUPPLEMENTARY FIGURES AND FIGURE LEGENDS

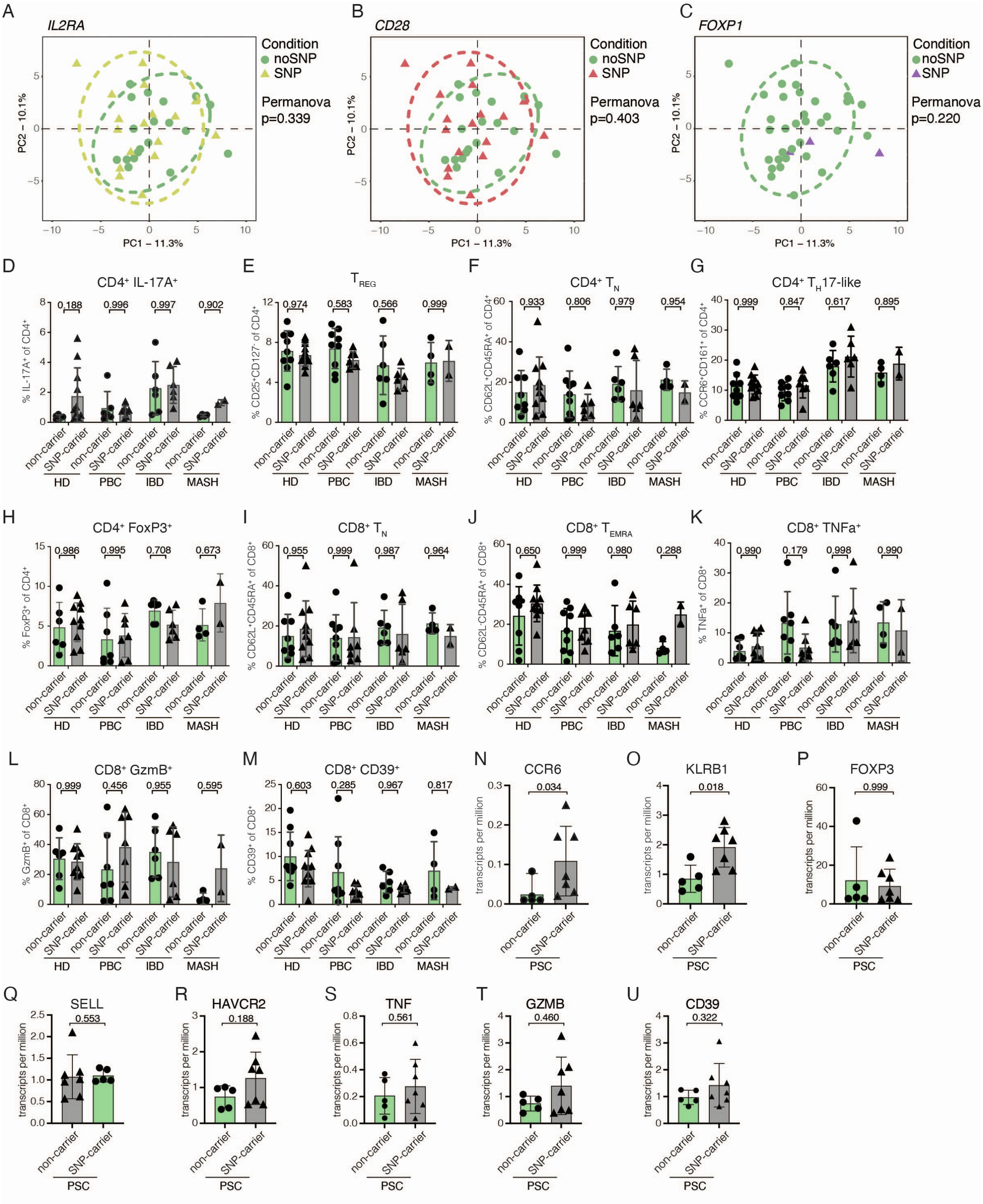

Fig. S1: People with PSC carrying *rs56258221* show distinct immunophenotypes. Related to Figure 1.

(**A-C**) Principal component analyses of the analyzed immunophenotyping data from peripheral blood of people with PSC, separated by the genotype for polymorphism *rs4147359* (*IL2RA*) (A), *rs7426056* (*CD28*) (B) and *rs80060485* (*FOXP1*) (C). (**D-H**) Frequencies of different CD4$^+$ T cell subsets. (**D**) T$_N$, identified by CD62L/CD45RA. (**E**) T$_H$17 cells identified by IL-17A expression upon stimulation with PMA/Ionomycin. (**F**)

T_H17 cells identified by CCR6/CD161. (**G**) T_{REG} identified by CD127/CD25. (**H**) T_{REG} identified by FoxP3 expression upon stimulation with PMA/Ionomycin. (**I**-**M**) Frequencies of different $CD8^+$ T cell subsets. (**I**) T_N, identified by CD62L/CD45RA. (**J**) T_{EMRA}, identified by CD62L/CD45RA. (**K**) CD39 expression on $CD8^+$ T cells. (**L**) TNFa expression upon stimulation with PMA/Ionomycin. (**M**) GzmB expression upon stimulation with PMA/Ionomycin. Statistics: Normality distribution was tested by Kolmogorov-Smirnov test; normal distribution: Welch's t-Test or 2-way ANOVA; no normal distribution: Mann-Whitney U Test. $p<0.05$ was considered statistically significant. Data is presented as mean ± SD and deriving from $n \geq 2$ repeats per experiment.

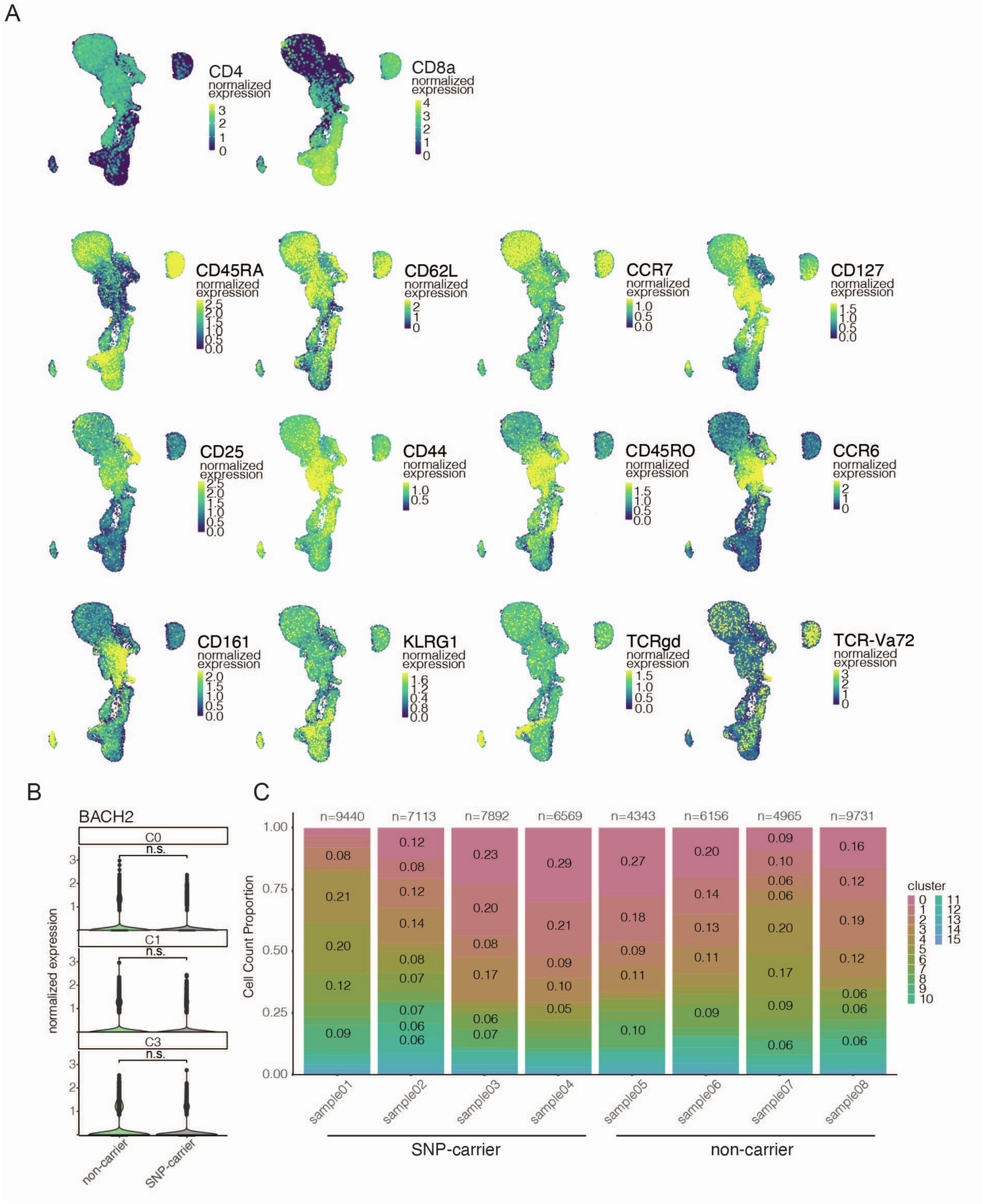

Fig. S2: Characterization of peripheral blood T cells from homozygous carriers of *rs56258221*. Related to Figure 2.

(**A**) Data of antibody-derived tags, resembling protein expression, mapped onto the UMAP from Fig. 2B. Differentiation markers were used to assist cluster assignment. (**B**) BACH2 mRNA expression levels in $CD4^{+}$ (C0, C1) and $CD8^{+}$ T_N (C3) between SNP-carriers and non-carriers. (**C**) Distribution of cells per sample across the different clusters of T cells between SNP-carriers and non-carriers.

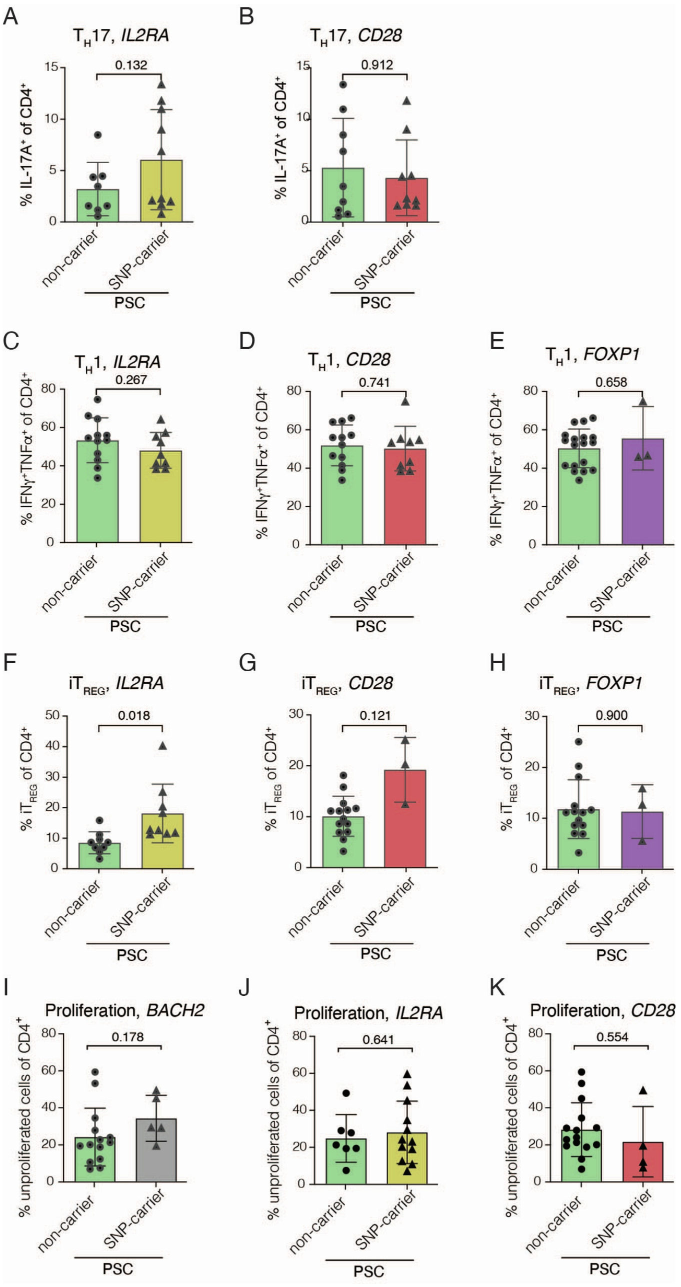

Fig. S3: Skewed differentiation of naive CD4$^+$ T cells from people with PSC is associated with *rs56258221*. Related to Figure 3.

(**A-B**) After 12 d of polarizing culture of CD4$^+$ T$_N$ from patients with PSC (n=18) the frequency of CD4$^+$IL-17A$^+$ cells was determined and separated by *rs4147359 (IL2RA)* (A)*, rs7426056 (CD28)* (B). *rs80060485 (FOXP1)* is not shown as no datapoints for SNP-carriers were recordfed. (**C-E**) After 7 d of polarizing culture of CD4$^+$ T$_N$ from patients with PSC (n=21), the frequency of T CD4$^+$CXCR3$^+$TBET$^+$IFNg$^+$TNFa$^+$ cells was determined and data was separated in to carriers and non-carriers of *rs4147359 (IL2RA)* (C)*, rs7426056 (CD28)* (D) and *rs80060485 (FOXP1)* (E). (**F-H**) After 7 days of polarizing culture of CD4$^+$ T$_N$ from patients with PSC (n=18), the frequencies of CD4$^+$CD25$^+$CD152$^+$FOXP3$^+$ cells were determined and data was separated into

carriers and non-carriers of *rs4147359 (IL2RA)* (F)*, rs7426056 (CD28)* (G) and *rs80060485 (FOXP1)* (H). (**I-K**) After 6 d of non-polarizing culture of *CellTrace Violet*-marked CD4$^+$ T$_N$ from patients with PSC, the frequency of divided and undivided cells was determined by flow cytometry. Data was separated into carriers and non-carriers of *rs56258221* (*BACH2*) *rs4147359* (*IL2RA*) (J)*, rs7426056* (*CD28*) (K). Data is presented as mean ± SD and deriving from n ≥ 2 repeats per experiment.

SUPPLEMENTARY TABLES

Table S1: Clinical parameters of people with PSC, related to Figure 1A-M.

patient #	SNP-status	gender	year of birth	IBD status	other autoimmune disease	immunosuppr. therapy	transient elastography [kPa]	MELD-score	ANAs (Ref.: <1:80)	SMA (Ref.: <1:80)	SLA/LP (Ref.: <20relU/ml)
**patient #1**	non-carrier	cis	1967	PSC-assoc. colitis	/	/	10	6,0	negative	negative	negative
**patient #2**	non-carrier	cis	1968	PSC-assoc. colitis	Hashimoto's disease	/	8,8	7,1	1:80	negative	negative
**patient #3**	non-carrier	cis	1983	/	/	/	4,5	6,8	negative	negative	negative
**patient #4**	non-carrier	cis	1957	PSC-assoc. colitis	/	/	3,9	6,0	1:80	negative	negative
**patient #5**	non-carrier	cis	1968	/	/	/	5,3	6,0	negative	n.a.	n.a.
**patient #6**	non-carrier	cis	1982	PSC-assoc. colitis	/	/	60,4	33,4	1:640	negative	negative
**patient #7**	non-carrier	cis	1968	PSC-assoc. colitis	/	/	4,7	6,0	1:80	negative	negative
**patient #8**	non-carrier	cis	1965	/	/	/	16,5	15,8	1:160	negative	negative
**patient #9**	non-carrier	cis	1955	/	/	/	6,3	6,0	negative	negative	negative
**patient #10**	non-carrier	cis	1959	/	/	/	5,7	6,0	1:80	negative	negative
**patient #11**	non-carrier	cis	1965	PSC-assoc. colitis	/	Azathioprine	32	6,9	1:640	1:160	negative
**patient #12**	non-carrier	cis	1967	PSC-assoc. colitis	/	/	4,9	6,7	1:320	negative	negative
**patient #13**	non-carrier	cis	1978	PSC-assoc. colitis	Graves' disease	/	4,7	6,0	1:160	1:80	negative
**patient #14**	non-carrier	cis	1977	PSC-assoc. colitis	/	Azathioprine	4	6,2	negative	negative	negative
**patient #15**	non-carrier	cis	1974	PSC-assoc. colitis	/	/	3,7	6,0	1:320	negative	negative
**patient #16**	non-carrier	cis	1982	PSC-assoc. colitis	/	/	4,3	6,0	1:320	negative	negative
**patient #17**	non-carrier	cis	1941	/	/	/	4,1	6,0	negative	negative	negative
**patient #18**	non-carrier	cis	1966	/	/	/	7,8	6,0	1:160	negative	negative
**patient #19**	heterozygous	cis	1950	PSC-assoc. colitis	/	/	7,6	6,0	1:1280	negative	negative
**patient #20**	heterozygous	cis	1979	PSC-assoc. colitis	/	/	34,4	13,0	1:5120	1:180	negative
**patient #21**	heterozygous	cis	1979	PSC-assoc. colitis	/	/	11,7	6,0	1:320	negative	negative
**patient #22**	heterozygous	cis	1953	PSC-assoc. colitis	/	/	3,5	6,0	1:320	negative	negative
**patient #23**	heterozygous	cis	1986	PSC-assoc. colitis	/	/	75	7,2	1:80	negative	negative
**patient #24**	heterozygous	cis	1978	PSC-assoc. colitis	/	/	4,4	6,0	1:320	1:320	negative
**patient #25**	heterozygous	cis	1958	/	/	/	n.a.	18,9	1:160	1:80	negative
**patient #26**	heterozygous	cis	1963	/	/	/	70	14,5	1:320	1:80	negative
**patient #27**	heterozygous	cis	1957	PSC-assoc. colitis	Hashimoto's disease	/	7,2	6,0	1:80	1:80	negative
**patient #28**	heterozygous	cis	1976	/	/	/	11,3	7,8	1:160	negative	negative
**patient #29**	heterozygous	cis	1969	PSC-assoc. colitis	DM type 1	/	6,6	7,7	1:320	1:160	negative
**patient #30**	heterozygous	cis	1952	PSC-assoc. colitis	/	/	n.a.	9,6	1:160	negative	negative
**patient #31**	heterozygous	cis	1977	PSC-assoc. colitis	/	/	4,8	6,0	1:180	negative	negative
**patient #32**	heterozygous	cis	1969	/	/	/	3,6	6,0	1:160	negative	negative
**patient #33**	heterozygous	cis	1956	Crohn's disease	Graves' disease	/	n.a.	21,1	1:640	negative	n.a.
**patient #34**	homozygous	cis	1957	PSC-assoc. colitis	/	/	7,1	7,5	1:640	negative	negative
**patient #35**	homozygous	cis	1969	PSC-assoc. colitis	/	/	6	6,8	1:1280	negative	negative
**patient #36**	homozygous	cis	1963	PSC-assoc. colitis	/	/	37,4	6,0	1:640	negative	negative

Table S2: Clinical parameters of people with PSC, related to Figure 2A-J.

patient #	SNP-status	gender	year of birth	IBD status	other autoimmune disease	immunosuppr. therapy	transient elastography [kPa]	MELD-Score	ANAs (Ref.: <1:80)	SMA (Ref.: <1:80)	SLA/LP (Ref.: <20relU/ml)
**patient #1**	homozygous	cis	1969	PSC-assoc. colitis	/	/	6	6,8	1:1280	negative	negative
**patient #2**	homozygous	cis	1987	/	/	/	20	11,8	1:320	negative	negative
**patient #3**	homozygous	cis	1980	PSC-assoc. colitis	/	Vedolizumab	5,6	6,0	negative	negative	negative
**patient #4**	homozygous	cis	1975	PSC-assoc. colitis	/	/	5,3	6,0	negative	1:80	negative
**patient #5**	non-carrier	cis	1966	/	/	/	7,8	6,0	1:160	negative	negative
**patient #6**	non-carrier	cis	1979	/	/	/	4,7	6,0	1:160	negative	negative
**patient #7**	non-carrier	cis	1976	/	/	/	6,3	6,0	1:160	negative	negative
**patient #8**	non-carrier	cis	1972	Crohn's disease	/	/	6,7	6,0	1:160	negative	negative

Table S3: Clinical parameters of people with PSC, related to Figures 2K-L and 4D.

patient #	SNP-status	gender	year of birth	IBD status	other autoimmune disease	immunosuppr. therapy	transient elastography [kPa]	MELD-Score	ANAs (Ref.: <1:80)	SMA (Ref.: <1:80)	SLA/LP (Ref.: <20relU/ml)
**patient #1**	non-carrier	cis	1966	/	/	/	7,8	6,0	1:160	negative	negative
**patient #2**	non-carrier	cis	1979	/	/	/	4,7	6,0	1:160	negative	negative
**patient #3**	non-carrier	cis	1976	/	/	/	6,3	6,0	1:160	negative	negative
**patient #4**	non-carrier	cis	1972	Crohn's disease	/	/	6,7	6,0	1:160	negative	negative
**patient #5**	non-carrier	cis	1980	PSC-assoc. colitis	/	/	7,8	6,0	1:160	negative	negative
**patient #6**	non-carrier	cis	1994	PSC-assoc. colitis	/	Azathioprin	4,9	6,0	1:320	1:80	negative
**patient #7**	non-carrier	cis	1975	PSC-assoc. colitis	Psoriasis vulgaris	Ustekinumab	6,3	6,0	1:160	negative	negative
**patient #8**	homozygous	cis	1944	PSC-assoc. colitis	/	/	14,3	14,1	1:160	1:80	negative
**patient #9**	homozygous	cis	1957	PSC-assoc. colitis	/	/	7,1	7,5	1:640	negative	negative
**patient #10**	homozygous	cis	1986	PSC-assoc. colitis	/	/	5,2	6,0	1:640	1:80	negative
**patient #11**	homozygous	cis	1972	PSC-assoc. colitis	/	/	43,8	14,5	1:320	negative	negative
**patient #12**	homozygous	cis	1963	PSC-assoc. colitis	/	/	37,4	6,0	1:640	negative	negative
**patient #13**	homozygous	cis	1969	PSC-assoc. colitis	/	/	6	6,8	1:1280	negative	negative
**patient #14**	homozygous	cis	1987	/	/	/	20	11,8	1:320	negative	negative
**patient #15**	homozygous	cis	1980	PSC-assoc. colitis	/	Vedolizumab	5,6	6,0	negative	negative	negative
**patient #16**	homozygous	cis	1975	PSC-assoc. colitis	/	/	5,3	6,0	negative	1:80	negative

Table S4: Clinical parameters of people with PSC, related to Figure 3H+K.

patient #	SNP-Status	gender	year of birth	IBD status	other autoimmune disease	immunosuppr. therapy	transient elastography [kPa]	MELD-score	ANAs (Ref.: <1:80)	SMA (Ref.: <1:80)	SLA/LP (Ref.: <20relU/ml)
**patient #1**	non-carrier	cis	1982	PSC-assoc. colitis	/	/	4,3	6,0	1:320	negative	negative
**patient #2**	non-carrier	cis	1977	PSC-assoc. colitis	/	/	6	6,0	1:80	1:80	negative
**patient #3**	non-carrier	cis	1967	PSC-assoc. colitis	/	/	4,9	6,7	1:320	negative	negative
**patient #4**	non-carrier	cis	1967	PSC-assoc. colitis	/	/	6,1	6,0	1:160	negative	negative
**patient #5**	non-carrier	cis	1980	PSC-assoc. colitis	/	/	2,1	9,9	1:640	negative	negative
**patient #6**	non-carrier	cis	1977	PSC-assoc. colitis	/	/	7,6	6,0	1:160	negative	negative
**patient #7**	non-carrier	cis	1972	PSC-assoc. colitis	/	/	23	6,0	negative	negative	negative
**patient #8**	non-carrier	cis	1987	/	/	/	75	6,0	negative	negative	negative
**patient #9**	non-carrier	cis	1964	/	/	/	4,7	6,0	1:160	negative	negative
**patient #10**	non-carrier	cis	1975	PSC-assoc. colitis	/	Azathioprin	14	7,1	1:160	negative	23.0
**patient #11**	non-carrier	cis	1976	PSC-assoc. colitis	Hashimoto's disease	/	7,4	6,0	1:80	negative	negative
**patient #12**	heterozygous	cis	1964	PSC-assoc. colitis	/	/	4,8	6,9	1:320	negative	negative
**patient #13**	heterozygous	cis	1950	PSC-assoc. colitis	/	/	7,6	6,0	1:1280	negative	negative
**patient #14**	heterozygous	cis	1961	PSC-assoc. colitis	Rheumatoid arthritis	Baricitinib	12,9	6,0	1:160	negative	negative
**patient #15**	heterozygous	cis	1980	PSC-assoc. colitis	/	/	6,1	6,0	1:80	negative	negative
**patient #16**	heterozygous	cis	1953	PSC-assoc. colitis	/	/	3,5	6,0	1:320	negative	negative
**patient #17**	homozygous	cis	1963	PSC-assoc. colitis	/	/	37,4	6,0	1:640	negative	negative
**patient #18**	homozygous	cis	1994	PSC-assoc. colitis	/	/	71	6,6	1:640	1:160	negative
**patient #19**	homozygous	cis	1980	PSC-assoc. colitis	/	/	5,6	6,0	negative	negative	negative

Table S5: Clinical parameters of people with PSC, related to Figure 3G+J.

patient #	SNP-status	gender	year of birth	IBD status	other autoimmune disease	immunosuppr. therapy	transient elastography [kPa]	MELD-score	ANAs (Ref.: <1:80)	SMA (Ref.: <1:80)	SLA/LP (Ref.: <20relU/ml)
**patient #1**	non-carrier	cis	1997	Crohn's disease	/	/	7	8,3	1:160	negative	negative
**patient #2**	non-carrier	cis	1956	/	/	/	4,5	6,0	1:160	negative	negative
**patient #3**	non-carrier	cis	1962	PSC-assoc. colitis	/	/	5,6	6,0	1:80	1:160	negative
**patient #4**	non-carrier	cis	1980	PSC-assoc. colitis	/	/	2,1	9,9	1:640	negative	negative
**patient #5**	non-carrier	cis	1951	PSC-assoc. colitis	/	Vedolizumab	4,3	6,0	1:160	negative	negative
**patient #6**	non-carrier	cis	1974	PSC-assoc. colitis	/	/	3,7	6,0	1:320	negative	negative
**patient #7**	non-carrier	cis	1950	/	/	/	4,6	6,0	1:5120	negative	negative
**patient #8**	non-carrier	cis	1980	PSC-assoc. colitis	/	/	7,8	6,0	1:160	negative	negative
**patient #9**	non-carrier	cis	1987	PSC-assoc. colitis	/	/	5,8	6,0	1:640	1:160	negative
**patient #10**	non-carrier	cis	1987	PSC-assoc. colitis	/	/	6,1	6,0	1:160	negative	negative
**patient #11**	non-carrier	cis	1962	PSC-assoc. colitis	/	/	3,3	6,0	1:80	negative	negative
**patient #12**	heterozygous	cis	1999	PSC-assoc. colitis	/	Fligotinib	4,7	6,0	1:80	negative	negative
**patient #13**	heterozygous	cis	1977	PSC-assoc. colitis	/	/	4,8	6,0	1:180	negative	negative
**patient #14**	heterozygous	cis	1966	/	Hashimoto's disease	/	4,2	6,0	1:160	negative	negative
**patient #15**	heterozygous	cis	1988	PSC-assoc. colitis	/	/	7,8	6,0	1:160	negative	negative
**patient #16**	heterozygous	cis	1968	PSC-assoc. colitis	/	/	42	16,0	1:5120	negative	n.a.
**patient #17**	heterozygous	cis	1957	/	/	/	6,6	6,0	1:160	negative	negative
**patient #18**	heterozygous	cis	1976	PSC-assoc. colitis	/	/	4,9	6,0	1:80	negative	negative
**patient #19**	heterozygous	cis	2000	Crohn's disease	/	Ustekinumab	3,7	6,0	1:1280	negative	negative
**patient #20**	heterozygous	cis	1969	/	/	/	3,6	6,0	1:160	negative	negative
**patient #21**	heterozygous	cis	1977	/	/	/	4,9	7,5	1:160	negative	negative

Table S6: Clinical parameters of people with PSC, related to Figure 3F+I.

patient #	SNP-status	gender	year of birth	IBD status	other autoimmune disease	immunosuppr. therapy	transient elastography [kPa]	MELD-score	ANAs (Ref.: <1:80)	SMA (Ref.: <1:80)	SLA/LP (Ref.: <20relU/ml)
**patient #1**	non-carrier	cis	1988	PSC-assoc. colitis	/	/	28	6,0	1:80	1:160	negative
**patient #2**	non-carrier	cis	1961	Crohn's disease	Hashimoto's disease	/	4,7	6,0	1:320	negative	negative
**patient #3**	non-carrier	cis	1977	PSC-assoc. colitis	Hashimoto's disease	/	10,4	7,4	1:640	negative	negative
**patient #4**	non-carrier	cis	1963	/	/	/	6,2	6,0	1:80	1:80	negative
**patient #5**	non-carrier	cis	1979	/	/	/	4,7	6,0	1:160	negative	negative
**patient #6**	non-carrier	cis	1988	Crohn's disease	/	Vedolizumab	8,8	12,8	1:640	negative	negative
**patient #7**	heterozygous	cis	1967	/	/	/	9,3	6,0	1:160	negative	negative
**patient #8**	heterozygous	cis	1975	PSC-assoc. colitis	/	/	7,1	6,0	1:160	negative	negative
**patient #9**	heterozygous	cis	1976	/	/	/	7,4	6,0	1:320	negative	negative
**patient #10**	heterozygous	cis	1976	PSC-assoc. colitis	/	/	4,9	6,0	1:80	negative	negative
**patient #11**	heterozygous	cis	1950	/	/	/	9,3	6,0	1:160	negative	negative
**patient #12**	heterozygous	cis	1950	PSC-assoc. colitis	/	/	12,7	7,2	1:320	negative	negative
**patient #13**	heterozygous	cis	1965	/	/	/	6,7	6,0	1:160	negative	negative
**patient #14**	heterozygous	cis	1965	/	/	/	5	6,0	1:320	negative	negative
**patient #15**	heterozygous	cis	1959	PSC-assoc. colitis	/	/	12,3	6,0	1:5120	negative	negative
**patient #16**	homozygous	cis	1963	PSC-assoc. colitis	/	/	37,4	6,0	1:640	negative	negative
**patient #17**	homozygous	cis	1944	PSC-assoc. colitis	/	/	14,3	14,1	1:160	1:80	negative

*Abbreviations:

n.a.: information not available

/ : patient without IBD; no immunosuppressive therapy; absence of tested autoantibodies

Table S7: Primers for generation of BACH2 plasmid, related to Figure 5F-H.

Primer	Sequence	Gene
Pr1	CACAC<u>GAGCTC</u>GATACTGTACAGTAATGG	Forward primer (3'UTR BACH2-mRNA)
Pr2	CACAC<u>GTCGAC</u>AAAGCAAAGAATATACAG	Reverse primer (3'UTR BACH2-mRNA)
Fr1	(SacI)<u>GAGCTC</u>GATACTGTACANCCACTGGTCAACTTTGCCACTTGCA<br>CTGAGTTTTGGGT**CAAACCTA**TTTTCTTAAATGAAGTTGTAACTTCG<br>GTATAACTCAAGTATACTGTATATTCTTTGCTTT<u>GTCGAC</u>(SalI)	BACH2 3'UTR fragment (miR4464 site in bold letters)
Pr4	GAAAATACTCGAGACCCAAAACTCAGTGC	Reverse primer (to mutate the miR4464 site)
Fr2	(SacI)<u>GAGCTC</u>GATACTGTACANCCACTGGTCAACTTTGCCACTTGCA<br>CTGAGTTTTGGGT**CTCGAGTA**TTTTCTTAAATGAAGTTGTAACTTCG<br>GTATAACTCAAGTATACTGTATATTCTTTGCTTT<u>GTCGAC</u>(SalI)	Mutated miR-4464 site in BACH2 3'-UTR (mutated part in bold letters)
